# Effect of Substituted Pyridine Co-Ligands and (Diacetoxyiodo)benzene Oxidants on the Fe(III)-OIPh-Mediated Triphenylmethane Hydroxylation Reaction

**DOI:** 10.3390/molecules29163842

**Published:** 2024-08-13

**Authors:** Patrik Török, József Kaizer

**Affiliations:** Research Group of Bioorganic and Biocoordination Chemistry, University of Pannonia, H-8201 Veszprém, Hungary; patriktrk6@gmail.com

**Keywords:** non-heme models, iron(III)-iodosylarene complex, alkane hydroxylation, oxidation, catalysis

## Abstract

Iodosilarene derivatives (PhIO, PhI(OAc)_2_) constitute an important class of oxygen atom transfer reagents in organic synthesis and are often used together with iron-based catalysts. Since the factors controlling the ability of iron centers to catalyze alkane hydroxylation are not yet fully understood, the aim of this report is to develop bioinspired non-heme iron catalysts in combination with PhI(OAc)_2_, which are suitable for performing C-H activation. Overall, this study provides insight into the iron-based ([Fe^II^(PBI)_3_(CF_3_SO_3_)_2_] (**1**), where PBI = 2-(2-pyridyl)benzimidazole) catalytic and stoichiometric hydroxylation of triphenylmethane using PhI(OAc)_2_, highlighting the importance of reaction conditions including the effect of the co-ligands (*para*-substituted pyridines) and oxidants (*para*-substituted iodosylbenzene diacetates) on product yields and reaction kinetics. A number of mechanistic studies have been carried out on the mechanism of triphenylmethane hydroxylation, including C-H activation, supporting the reactive intermediate, and investigating the effects of equatorial co-ligands and coordinated oxidants. Strong evidence for the electrophilic nature of the reaction was observed based on competitive experiments, which included a Hammett correlation between the relative reaction rate (log*k*_rel_) and the σ_p_ (4R-Py and 4R’-PhI(OAc)_2_) parameters in both stoichiometric (ρ = +0.87 and +0.92) and catalytic (ρ = +0.97 and +0.77) reactions. The presence of [(PBI)_2_(4R-Py)Fe^III^OIPh-4R’]^3+^ intermediates, as well as the effect of co-ligands and coordinated oxidants, was supported by their spectral (UV–visible) and redox properties. It has been proven that the electrophilic nature of iron(III)-iodozilarene complexes is crucial in the oxidation reaction of triphenylmethane. The hydroxylation rates showed a linear correlation with the Fe^III^/Fe^II^ redox potentials (in the range of −350 mV and −524 mV), which suggests that the Lewis acidity and redox properties of the metal centers greatly influence the reactivity of the reactive intermediates.

## 1. Introduction

Catalytic oxidation of alkanes under mild conditions is quite difficult due to their high C-H bond energy. However, these reactions can be catalyzed by various metalloenzymes such as cytochrome P-450, methane monooxygenase, and Rieske dioxygenase, based on which numerous biomimetic oxidation reactions have also been developed [1,2,3,4,5,6,7]. Several efficient biomimetic oxidation reactions have been developed using iron complexes as catalysts with various single oxygen donor oxidants such as PhIO, H_2_O_2,_ or ROOH [8,9,10,11]. Oxidative functionalization of alkanes into more valuable products (e.g., alcohols, ketones, aldehydes) is of great importance nowadays [12,13,14,15,16,17,18,19]. Complexes of manganese and iron with various ligands such as porphyrins, tetraazaannulenes, phthalocyanines, chlorines, Schiff bases, and triazacyclononanes have been extensively studied as alkane oxidation catalysts [12,20,21,22,23,24,25,26,27,28].

Although great efforts have been made to fine-tune the reactivity of iron–oxo complexes in oxidation reactions by modifying the structural and electronic properties of the metal center, there are few studies in the literature on other reactive intermediates such as Fe^III^-OIPh, Fe^III^-OOH, and Fe^III^-OOR species. In many cases, due to their Janus face, these intermediates not only participate in reactions as precursor complexes of Fe^IV^ = O or Fe^V^ = O intermediates but can also participate in oxidation processes as active species [29,30,31,32,33,34,35,36,37,38,39,40,41,42,43,44]. Since the redox properties of catalysts and their intermediates play a special role in redox reactions, such as in hydrogen atom transfer (HAT) and oxygen atom transfer (OAT), it is therefore important to examine what factors influence the reactivity of these complexes. In the case of high valence metal oxo species, it has been shown that the reactivity can be influenced not only by the oxidation and spin state of the metal center(s) but also by the nature of the supporting ligands, including the axial and equatorial co-ligands. [45,46,47,48,49,50,51,52,53,54,55,56,57]. Terminal oxidants, such as the insoluble, polymeric PhIO and the organic solvent-soluble PhI(OAc)_2_, can be used as mild oxidants in many metal complex-catalyzed oxygenation processes, where their main role is the formation of high-valent metal–oxo intermediates, but in many cases the formation and active role of Fe^III^-OIPh complexes in the oxidation reactions was also confirmed [29,30,31,32,33,42,43,44]. 

Previous studies have shown that iron(II) complexes with PBI-type ligands (PBI = 2-(2-pyridyl)benzimidazole) can be used as precursors to produce a number of reactive intermediates believed to be involved in iron-based oxidation processes. With their help, both the [(PBI)_4_(Solvent)_2_Fe^III^_2_(μ-O_2_)]^4+^ [58,59,60,61,62] and the [(PBI)_2_(Solvent or additives)Fe^III^(OIPh)]^3+^ intermediates can be synthesized by the use of H_2_O_2_, and PhIO (or PhI(OAc)_2_), respectively [42,43,44]. The structure of the above intermediates was assumed based on UV–VIS, EPR, and rRaman measurements [43]. Thanks to the ligands, the advantage of these systems is that both the iron(II) precursors and the formed high-valent intermediates have favorable thermal stability, enabling their detailed characterization, the investigation of their spectral and redox properties, and the effect of these characteristics on their reactivity. Considering the results so far, it can be said that the above complexes are suitable as catalysts for the oxygenation of thioanisoles and other compounds containing C-H and C=C bonds with PhIO or its more soluble and mild oxygen source derivative, PhI(OAc)_2_, or deformylation of various aldehydes in a nucleophilic reaction with H_2_O_2_. As a result of previous catalytic studies, it was established that in the case of OAT reactions, such as sulfoxidation and epoxidation, the yield of the reactions can be significantly increased if monodentate pyridines with electron-withdrawing groups are used as equatorial ligands for the catalytically active [(PBI)_2_(4R-Py)Fe^III^(OIPh)]^3+^. Based on the detailed mechanistic studies, the linear free energy (Hammett equations) relationships through the positive ρ-value obtained for the co-ligands indicate that the reactive intermediate of the above reactions is electrophilic [44].

**Scheme 1:**
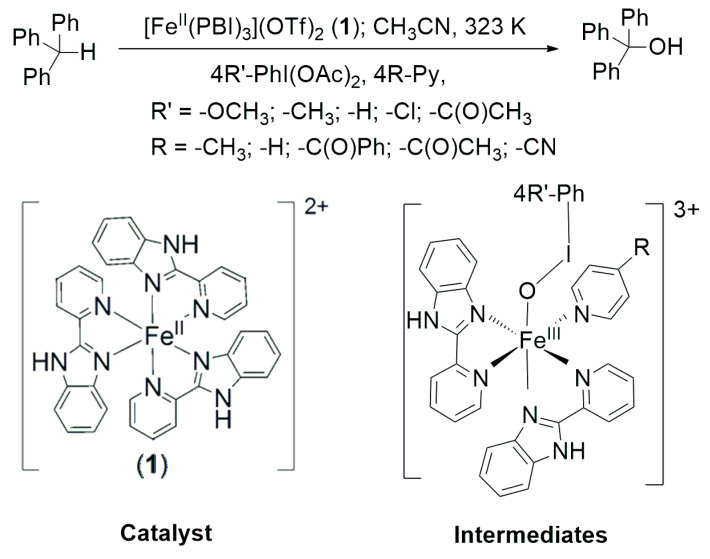
[Fe^II^(PBI)_3_](OTf)_2_ (**1**) catalyzed oxidation of triphenylmethane mediated by [(PBI)_2_(4R-Py)Fe^III^(4R’-PhIO)]^3^+ intermediates in the presence of 4R’-PhI(OAc)_2_ terminal oxidants and 4R-Py co-ligands.

The aim of this report is to study the catalytic and stoichiometric oxidation reactions involving C-H activation using the sample substrate triphenylmethane, namely to study the effect of monodentate 4R-pyridine ligands and 4R’-PhIO (from 4R’-PhI(OAc)_2_) terminal oxidants with electron-withdrawing and electron donor substituents (Scheme 1). Our further aim is to compare the HAT (this study) and the previously published processes [44] mediated by [(PBI)_2_(4R-Py)Fe^III^(OIPh)]^3+^ intermediates based on the kinetic and catalytic results.

## 2. Results and Discussion

Based on previously published results, the complex, [Fe^II^(PBI)_3_](OTf)_2_ (**1**) (OTf = CF_3_SO_3_), catalyzes the sulfoxidation of thioanisole and the epoxidation of cis-cyclooctene and cis- and trans-stilbene with PhI(OAc)_2_ as an oxidant, where the optimal catalytic conditions were as follows: 1:100:300 ratio for catalyst–oxidant–substrate, in acetonitrile at 323 K [62]. Under these conditions, we achieved a conversion value of 35% for thioanisole, 22.46% for cis-cyclooctene, 24.14% for cis-stilbene, and 36.7% for trans-stilbene in the oxygen atom transfer (OAT) processes. Another observation was that the above values could be further increased by adding pyridine ([1]:[Py] = 1:10) as a possible equatorial ligand, namely 81.79%, 36.94%, 36.12%, and 41.15% conversion values were observed for thioanisole, cis-cyclooctene, and cis- and trans-stilbene, respectively, after 4 h. Hammett correlations between relative reaction rates (logk_rel_) and the σ_p_ (4R-Py) parameters, where ρ = +0.16, +0.95, +0.59, and +0.46 for thioanisole, cis-cyclooctene, and cis- and trans-stilbene, respectively, imply the electrophilic character of the OIPh group of the Fe^III^(OIPh) intermediate and positive charge buildup on the substrates. This proved crucial in controlling the nature of the active oxidizing form, which significantly affected its reactivity in OAT reactions [62]. Based on these results, an electrophilic oxidant, most likely a [(PBI)_2_(4R-Py)Fe^III^OIPh]^3+^ intermediate, can be assumed as a result of the reaction of **1** with PhIO (PhI(OAc)_2_) and then with pyridine derivatives. These species show characteristic spectral features (λ_max_ = 700–760 nm, ε = ~1400 M^−1^ cm^−1^ with S = ½ low-spin state) [43,45,62]. 

### 2.1. Effect of Equatorial Ligands on the Fe^III^OIPh-Mediated Oxidation of Triphenylmethane

**Figure 1:**
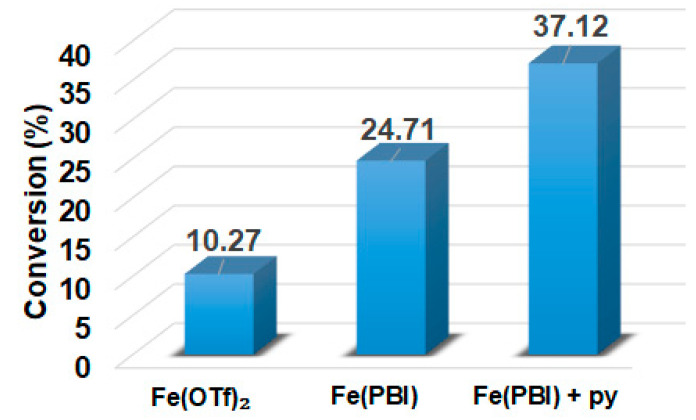
[Fe^II^(PBI)_3_](OTf)_2_-catalyzed hydroxylation of triphenylmethane with PhI(OAc)_2_ in the absence and presence of *para*-substituted pyridines in acetonitrile at 323 K: [Fe^II^(OTf)_2_]_0_ = 1 × 10^−3^ M, [**1**]_0_ = 1 × 10^−3^ M, [PhI(OAc)_2_]_0_ = 1 × 10^−1^ M, and [Ph_3_CH]_0_ = 3 × 10^−1^ M, [pyridine]_0_ = 1 × 10^−2^ M.

**Figure 2:**
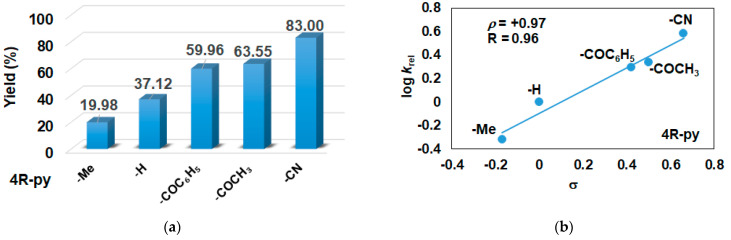
[Fe^II^(PBI)_3_](OTf)_2_-catalyzed hydroxylation of triphenylmethane with PhI(OAc)_2_ in the presence of *para*-substituted pyridines in acetonitrile at 323 K: (**a**) the calculated conversion (=TON) values for *para*-substituted pyridines. (**b**) Hammett plot of log*k*_rel_ against the *σ*_p_ of *para*-substituted pyridines. [**1**]_0_ = 1 × 10^−3^ M, [PhI(OAc)_2_]_0_ = 1 × 10^−1^ M, [Ph_3_CH]_0_ = 3 × 10^−1^ M, [pyridine]_0_ = 1 × 10^−2^ M.

In this report, we wanted to investigate the effect of equatorial pyridines (4R-py; R = -CH_3_, -H, -C(O)C_6_H_5_, -C(O)CH_3_, -CN) and the oxidant 4R’-PhI(OAc)_2_ (R = -OCH_3_, -CH_3_, H, -Cl, -C(O)CH_3_) in catalytic oxidation processes that involve C-H activation, using **1** as catalyst and triphenylmethane as model substrate. In order to compare the systems with OAT and C-H activation, we used the previously observed optimal conditions with a catalyst–additive–oxidant–substrate ratio of 1:10:100:300, in acetonitrile at 323 K for 4 h [62]. The catalyst–co-ligand ratio was chosen to be 1:10 based on the maximum shift of the characteristic λ_max_ of the Fe^III^(OIPh) species due to pyridine additives [62]. As a mild oxygen source, PhI(OAc)_2_ has the advantage of not damaging the catalysts and showing no noticeable reactivity with the substrate [63]. According to our previous studies, the formation of the more oxidizing PhIO can be explained by the reaction of PhI(OAc)_2_ with a trace amount of water [64]. Based on the blank experiments, it can be said that no hydroxylated products were formed in the absence of either the catalyst or PhI(OAc)_2_. At first glance, it is clear that the catalytic activity of the iron salt [Fe^II^(CH_3_CN)_4_(OTf)_2_] is negligible (conversion = 10.27%). In the case of [Fe^II^(PBI)_3_](OTf)_2_ (**1**), a 2.5-fold increase in activity was observed with a conversion value of 24.71% (TOF = 6.17 h^−1^) (Figure 1). The results of the comprehensive screening of [Fe(PBI)_3_](OTf)_2_ with and without pyridines are shown in Table 1. As expected, triphenylmethanol was the only oxidized product without any other byproducts after 4 h. Based on the obtained conversion values, it can be concluded that the pyridine derivatives have a significant effect on the test reactions. In the case of pyridine (**1**:py = 1:10), the conversion value increased by 1.5 times (37.12%; TOF = 9.28 h^−1^). The effect of electron-donating (-CH_3_) and electron-withdrawing (-CN, -C(O)C_6_H_5_, -C(O)CH_3_) substituents of pyridines on the reactivity was also investigated in competitive experiments, and a significant effect on catalytic oxidation of triphenylmethane was shown (Table 1 and Figure 2a), where the 4-CN-pyridine resulted in the highest (83.00%, TOF = 20.75 h^−1^), and 4-Me-pyridine gave the lowest conversion with 19.98% (TOF = 4.99 h^−1^). The effect is remarkable, which can be attributed to the increased electrophilicity of the ([(PBI)_2_(4R-Py)Fe^III^OIPh]^3+^) species due to the electron-withdrawing pyridine ligand.

The relative rates (k_rel_) for the [Fe^II^(PBI)_3_](OTf)_2_-catalyzed hydroxylation of triphenylmethane in the presence of para-substituted pyridines (4R-Py, R = -CH_3_, H, -C(O)C_6_H_5_, -C(O)CH_3_ and CN) was determined by measuring the formation of triphenylmethanol by GC. Figure 2b depicts a linear correlation (R = 0.96) of log k_rel_ versus Hammett σ_p_ constants in the competitive [Fe^II^(PBI)_3_](OTf)_2_-catalyzed hydroxylation reaction. The slope (ρ) of the plot is +0.97, clearly indicating that the electron-deficient intermediates are much more reactive than the electron-rich species, which is consistent with the involvement of an electrophilic oxidant, probably a putative [(PBI)_2_(4R-Py)Fe^III^OIPh]^3+^ intermediate. 

Summarizing the results of the competitive experiments and comparing them with the results of the sulfoxidation and epoxidation experiments, it can be said that the value obtained for co-ligands (ρ = +0.97) is much higher than the value obtained for sulfoxidation (ρ = +0.16), which indicates that the oxidation reaction involving C-H activation is much more sensitive to the electrophilic nature of the intermediate as the sulfoxidation reaction described by the direct oxygen transfer (DOT) mechanism [62]. Considering the previous results of the oxidation of cis-cyclooctene (ρ = 0.95) and *cis*- and *trans*-stilbene (ρ = 0.59 and +0.46, respectively) under the same conditions, it can be said that both the C-H activation and the epoxidation with nonconcerted ET mechanism are greatly influenced by the electrophilic character of the reactive intermediate (oxidant) [44]. Unfortunately, hydrocarbons with a higher BDE value (such as PhCH_3_) did not give the reaction under the given conditions.

### 2.2. Effect of Co-Oxidant (4R’-PhI(OAc)_2_) on the Fe^III^OIPh-Mediated Oxidation of Triphenylmethane

**Figure 3:**
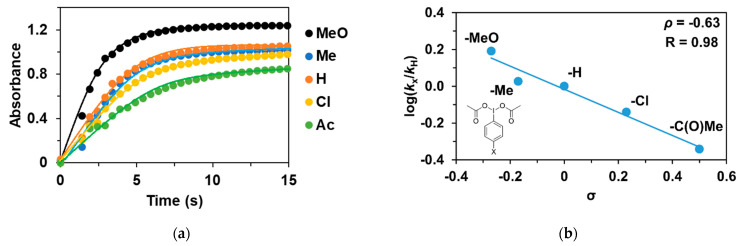
(**a**) Formation of [(PBI)_2_(MeCN)Fe^III^(4R-PhIO)]^3+^ intermediates in the in situ reaction of **1** with 4R’-Ph(IOAc)_2_ in acetonitrile at 293 K monitored at 760 nm. (**b**) Hammett plot of log*k*_rel_ against the *σ*_p_ of *para*-substituted 4R’-Ph(IOAc)_2_ oxidants. [**1**]_0_ = 1 × 10^−3^ M, [4R’-PhI(OAc)_2_]_0_ = 1 × 10^−2^ M.

As noted earlier, the reaction of **1** with PhIO leads to reactive Fe^III^(OIPh) formations, suggesting that it may play a key role in the catalytic cycles. This is also supported by the UV–Vis spectrum of the catalytic reaction mixture, where the rise and fall of their characteristic chromophores at λ_max_ = 700–760 nm (depending on the pyridines used as co-ligand) is clearly visible. After that, we wanted to investigate the effect of the electron-donating and electron-withdrawing groups introduced into the co-oxidant (4R’-PhI(OAc)_2_, where R’ = -OCH_3_, -CH_3_, -H, -Cl, and -C(O)CH_3_) on the catalytic oxidation of triphenylmethane with **1**. Before the detailed investigation of the catalytic system, we studied the formation kinetics of Fe^III^OIPh-4R’ adducts, with special attention paid to the role of the substituents on the co-oxidant. The rates in the presence of excess of PhI(OAc)_2_ (10 equivalent) obeyed pseudo-first-order kinetics. The pseudo-first-order rate constants, k_obs_, in the reaction of **1** with 4R-PhI(OAc)_2_ (R’ = -OCH_3_, -CH_3_, -H, -Cl, and -C(O)CH_3_) were determined from the absorbance change at 760 nm (Table 2 and Figure 3a). Based on the obtained data, the fastest adduct formation was observed for 4R-PhI(OAc)_2_ containing an electron-donating substituent (0.637 s^−1^ for -OCH_3_), while for the electron-withdrawing group -C(O)Me was the slowest (0.187 s^−1^). The observed kinetic data were treated based on the Hammett equation, from which the value of ρ was −0.63 (Figure 3b). Since the formation of the adduct in the case of PhIO is orders of magnitude faster than in the case of PhI(OAc)_2_, based on the negative ρ-value, it can be assumed that the rate-determining step is the formation of PhIO by the proton-assisted hydrolysis of PhI(OAc)_2_, which means that the higher electron density of the I(OAc)_2_ group increases the reaction rate. 

To investigate the effect of the oxidant, the catalytic oxidation of triphenylmethane was carried out with **1** and 4R’-PhI(OAc)_2_ (R’ = -OCH_3_, -CH_3_, -H, -Cl, and -C(O)CH_3_) derivatives under the conditions used for the study of the effect of co-ligands earlier (catalyst–oxidant–substrate ratio is 1:100:300 in acetonitrile at 323 K).

**Figure 4:**
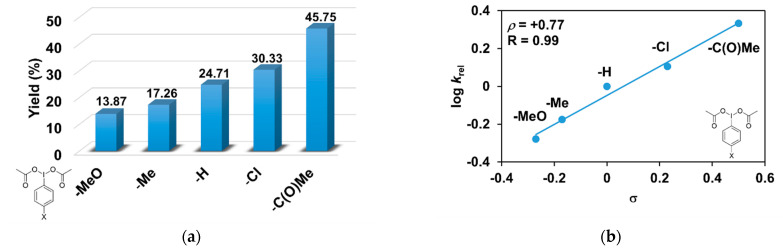
[Fe^II^(PBI)_3_](OTf)_2_-catalyzed hydroxylation of triphenylmethane with 4R’-PhI(OAc)_2_ in acetonitrile at 323 K: (**a**) the calculated conversion (=TON) values for 4R’-PhI(OAc)_2_. (**b**) Hammett plot of log*k*_rel_ against the *σ*_p_ of 4R-PhI(OAc)_2_. [**1**]_0_ = 1 × 10^−3^ M, [4R’-PhI(OAc)_2_]_0_ = 1 × 10^−1^ M, and [Ph_3_CH]_0_ = 3 × 10^−1^ M.

As evident in Table 3, the catalytic oxidation of triphenylmethane was affected by the substituents on the oxidant, 4R’-PhI(OAc)_2_ used. For example, the 4-C(O)CH_3_-PhI(OAc)_2_ resulted in the highest (45.75%), and the 4- CH_3_O-PhI(OAc)_2_ gave the lowest conversion value with 13.87% for the hydroxylation of triphenylmethane (Figure 4a). The effect is also remarkable and can be traced back to the increased electrophilicity of the [(PBI)_2_(CH_3_CN)Fe^III^OIPh-4R’]^3+^ species, due to the electron-withdrawing substituents of the PhIO oxidant, which is consistent with the results obtained for pyridine co-ligands. 

The relative rates (k_rel_) for the [Fe^II^(PBI)_3_](OTf)_2-_catalyzed hydroxylation of triphenylmethane with substituted iodosobenzene diacetates (4R-PhI(OAc)_2_, R = -OCH_3_, -CH_3_, -H, -Cl, and -C(O)CH_3_) was determined by measuring the formation of triphenylmethanol by GC. Figure 4b shows a linear correlation (R = 0.99) of log k_rel_ versus Hammett σ_p_ constants in the competitive reactions (Figure 4b). The slope (ρ) of the plot is +0.77, which means that the reaction of electron-deficient intermediates is much more favored. This value is similar to the value obtained for substituted pyridines (ρ = +0.97), which confirms the importance of the electrophilic feature of the [(PBI)_2_(CH_3_CN)Fe^III^OIPh-4R’]^3+^ intermediate. 

### 2.3. Effect of Equatorial Ligands on the Fe^III^OIPh-Mediated Stoichiometric Oxidation of Triphenylmethane

As a possible elementary step of the catalytic reaction, we wanted to investigate the stoichiometric oxidation of the in situ formed iron(III)-iodosylbenzene adduct with triphenylmethane, with particular attention paid to the effect of pyridine co-ligands. Based on our previous results [62], for the full formation of [(PBI)_2_(4R-Py)Fe^IlI^OIPh]^3+^ (R = -CH_3_, -H, C(O)CH_3_, -C(O)Ph, and -CN) in the reaction of **1** with PhI(OAc)_2_, the optimal amount of co-ligands (10 equivalents) was determined by titration. The λ_max_ values of the [(PBI)_2_(4R-Py)Fe^IlI^OIPh]^3+^ complexes are shown in Table 4 [62]. The observed hypochromic shift in the near IR region clearly indicated the coordination of the pyridine ligands, as well as their electronic effect on the electrophilicity of the active species, based on the observed trend of the Hammett correlation between the λ_max_^−1^ (at 700–760 nm) and the Hammett constants (σ_p_) [62]. These results imply that the effect of the ligands appears in the redox properties of the intermediates, as well as in their redox potential values, which may be consistent with our previous spectroscopic results.

**Figure 5:**
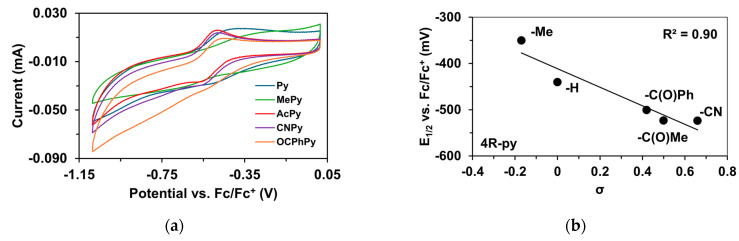
Redox properties of the [(PBI)_2_Fe^III^(OIPh)(4R-Py)] intermediates generated in situ by the reaction of **1** with Ph(IOAc)_2_ in acetonitrile at 293 K. (**a**) Cyclic voltammograms of [(PBI)_2_Fe^III^(OIPh)(4R-Py)] intermediates. (**b**) *E*_1/2_ vs. σ values for [(PBI)_2_[(4R-Py)Fe^III^(OIPh)]^3+^ intermediates. Conditions: [[Fe^II^(PBI)_3_](OTf)_2_]_0_ = 1 × 10^−3^ M, [PhI(OAc)_2_]_0_= 2 × 10^−3^ M, [4R-Py]_0_ = 1 × 10^−2^ M, in (0.1 M TBAClO_4_) CH_3_CN (10 cm^3^), scan rate: 1500 mV/s.

To determine the half-wave potentials (*E*_1/2_) of the in situ-generated [(PBI)_2_(4R-Py)Fe^IlI^OIPh]^3+^ (R = -CH_3_, -H, C(O)CH_3_, -C(O)Ph, and -CN) complexes, cyclic voltammetric measurements (CV) were also performed in acetonitrile. Cyclic voltammograms of [(PBI)_2_(4R-Py)Fe^IlI^OIPh]^3+^ show a single reversible one-electron oxidation wave with peak separations similar to ferrocene in the same solution (Table 5, Figure 5a). The peaks belong to the Fe^III^/Fe^II^ redox couple. By applying the Randles Sevcik equation, their reaction is found reversible and occurred under diffusion control system (Figure S1–S19) [65,66,67]. The half-wave potentials (*E*_1/2_) of the generated [(PBI)_2_(4R-Py)Fe^IlI^OIPh]^3+^ (R = -CH_3_, -H, C(O)CH_3_, -C(O)Ph, and -CN) complexes become more negative in the following sequence: R is 4-Me-Py (−350 mV) > Py (−440.4 mV) > 4-PhC(O)-Py (−500.6 mV) > 4-MeC(O)-Py (−523.2 mV) > 4-CN-Py (−524 mV). The *E*_1/2_ spans a 174 mV range from −524 mV (R = 4-CN-Py) to −350 mV (X = 4-Me-Py) vs. Fc/Fc^+^), indicating that replacement of the CH_3_CN with electron-rich pyridines results in a remarkable increase in the redox potential value. Furthermore, the *E*_1/2_ potentials correlate well with the Hammett σ_p_ constants of the 4R-Py (Figure 5b) as well as the λ_max_ values. 

**Figure 6:**
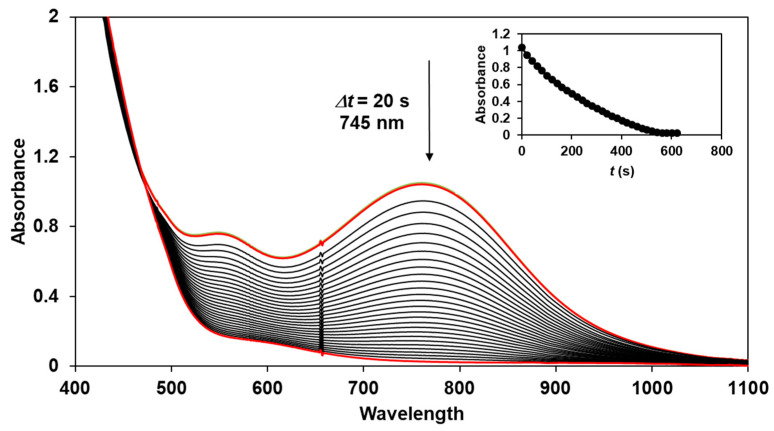
Decrease in absorbance of [(PBI)_2_[(4Ac-Py)Fe^III^(OIPh)]^3+^ intermediate in the stoichiometric oxidation of triphenylmethane at 293 K in acetonitrile. Inset: time course of the decay monitored at 745 nm. [**1**]_0_ = 1 × 10^−3^ M, [PhI(OAc)_2_]_0_ = 1.2 × 10^−3^ M, [triphenylmethane]_0_ = 1 × 10^−1^ M, [4Ac-Py]_0_ = 1 × 10^−2^ M.

**Figure 7:**
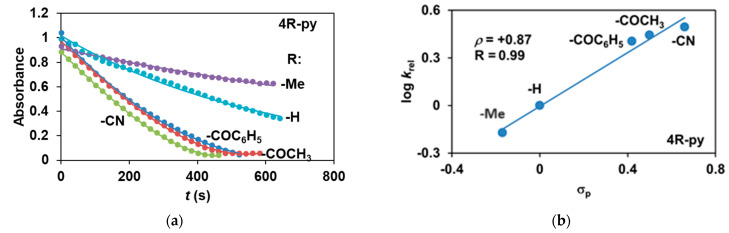
Stoichiometric hydroxylation of triphenylmethane with [(PBI)_2_(4R-Py)Fe^III^(OIPh)]^3+^ intermediates generated in situ by the reaction of **1** with Ph(IOAc)_2_ and 4R-Py derivatives in acetonitrile at 293 K: (**a**) monitoring the decrease in absorbance of [(PBI)_2_(4R-Py)Fe^III^(OIPh)]^3+^ intermediates by UV–Vis spectroscopy at 723–760 nm over time at 293 K. (**b**) Hammett plot of log*k*_rel_ against the *σ*_p_ of 4R-Py. [**1**]_0_ = 1 × 10^−3^ M, [PhI(OAc)_2_]_0_ = 1.2 × 10^−3^ M, [Ph_3_CH]_0_ = 1 × 10^−1^ M, [4R-Py]_0_ = 1 × 10^−2^ M.

**Figure 8:**
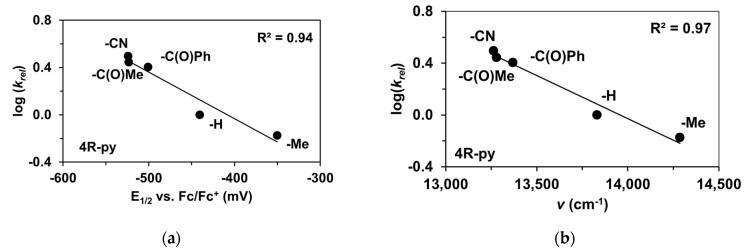
Stoichiometric hydroxylation of triphenylmethane with [(PBI)_2_(4R-Py)Fe^III^(OIPh)]^3+^ intermediates generated in situ by the reaction of [Fe^II^(PBI)_3_](OTf)_2_ with Ph(IOAc)_2_ and 4R-Py derivatives in acetonitrile at 293 K: (**a**) log(*k*_rel_) against *E*_1/2_ for [(PBI)_2_(4R-Py)Fe^III^(OIPh)]^3+^ intermediates. (**b**) log(*k*_rel_) against ν for [(PBI)_2_(4R-Py)Fe^III^(OIPh)]^3+^ intermediates. [**1**]_0_ = 1 × 10^−3^ M, [PhI(OAc)_2_]_0_ = 1.2 × 10^−3^ M, [Ph_3_CH]_0_ = 1 × 10^−1^ M, [4R-Py]_0_ = 1 × 10^−2^ M.

**Figure 9:**
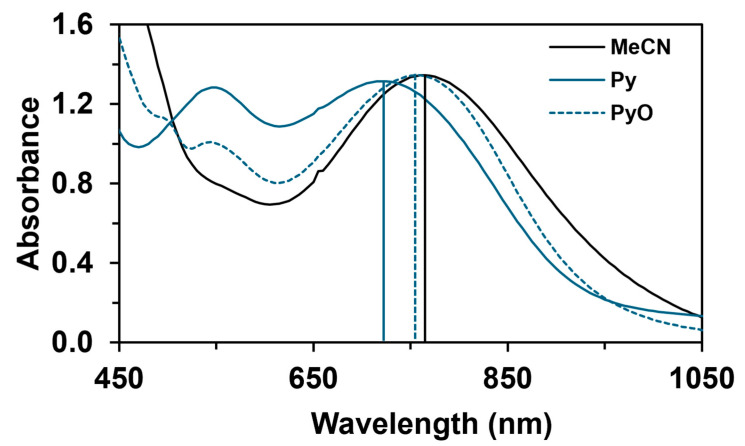
UV–Vis spectral properties of [(PBI)_2_[(X)Fe^III^(OIPh)]^3+^ (X = CH_3_CN, Py, PyO) intermediates at 293 K in acetonitrile. [**1**]_0_ = 1 × 10^−3^ M, [PhI(OAc)_2_]_0_ = 1.2 × 10^−3^ M, [triphenylmethane]_0_ = 1 × 10^−1^ M, [X]_0_ = 1 × 10^−2^ M.

The reactivity of the in situ-formed [(PBI)_2_(4R-Py)Fe^IlI^OIPh]^3+^ (R = -CH_3_, -H, C(O)CH_3_, -C(O)Ph, -CN) species was investigated in stoichiometric hydroxylation reactions with triphenylmethane. The reaction was carried out in CH_3_CN at 293 K with an excess of Ph_3_CH substrate, and the time courses for the decay of the in situ-generated [(PBI)_2_(4R-Py)Fe^IlI^OIPh]^3+^ intermediates were monitored at their characteristic near-IR wavelengths (λ_max_) in the range of 700–760 nm (Figure 6). The decay process resulted in the formation of triphenylmethanol as main products (Yield ~80%/[1] based on GC) without any side-products based on GC-Ms analysis. All reactions follow general second-order kinetics, in first-order Ph_3_CH and [(PBI)_2_(4R-Py)Fe^IlI^OIPh]^3+^ complexes (Figure 7a). Electron-releasing substituents in the pyridine co-ligand reduce the rate of oxidation. Correlations with σ (ρ = +0.87, Figure 7b) and with *E*_1/2_ (slope −3.94 V^−1^, Figure 8a and Figure S20–S28) and ν (Figure 8b) are evidence for an electrophilic mechanism. In the presence of PhIO and Py, the formation of pyridin-*N*-oxide (PyO) and its participation as a co-ligand in the reaction cannot be ruled out. When the stoichiometric reaction was performed in the presence of 10 equivalents of PyO, a much smaller hypsochromic shift (10 nm) and a nearly three-fold rate (*k*_obs_ = 3.14 × 10^−3^ s^−1^; (*k*_obs_ = *k*_selfdecay_ + *k*_2_[PH_3_CH] and *k*_obs_ >>> *k*_selfdecay_) compared to pyridine (*k*_obs_ = 1.33 × 10^−3^ s^−1^) were observed (Figure 9), consistent with a ν vs log*k_rel_* relationship (Table 4, Figure 8b).

**Figure 10:**
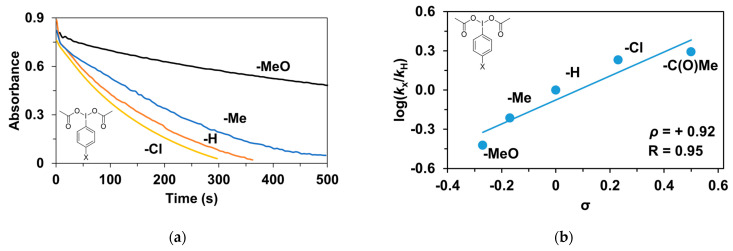
Stoichiometric hydroxylation of triphenylmethane with [(PBI)_2_(MeCN)Fe^III^(OIPh-4R)]^3+^ (R = -Cl, -H, -Me, -OMe) intermediates generated in situ by the reaction of **1** with 4R-Ph(IOAc)_2_ (R = -Cl, -H, -Me, -OMe) oxidants in acetonitrile at 293 K: (**a**) Monitoring the decrease in absorbance of [(PBI)_2_(CH_3_CN)Fe^III^(OIPh-4R)]^3+^ intermediates by UV–Vis spectroscopy at 760 nm over time at 293 K. (**b**) Hammett plot of log*k*_rel_ against the *σ*_p_ of [(PBI)_2_(CH_3_CN)Fe^III^(OIPh-4R)]^3+^intermediates. [[Fe^II^(PBI)_3_](OTf)_2_]_0_ = 1 × 10^−3^ M, [4R-PhI(OAc)_2_]_0_ = 1.2 × 10^−3^ M, [Ph_3_CH]_0_ = 1 × 10^−1^ M.

Finally, the tests were also extended to the effect of the oxidizing agent (PhI(OAc)_2_). *Para*-substituted (4R’-PhI(OAc)_2_, R’ = -CH_3_, -OCH_3_, -H, -Cl, -C(O)CH_3_) oxidants were used for the competition (Hammett correlation) stoichiometric reactions. The effect of the substituents of the 4R’-PhI(OAc)_2_ oxidants on the relative reactivity was studied for triphenylmethane and a remarkable effect on the stoichiometric hydroxylation reactions was observed (Table 6 and Figure 10a). Electron-withdrawing groups such as -C(O)CH_3_ gave significantly higher rate (*k*_obs_ = 9.22 × 10^−3^ s^−1^) than electron-donating groups such as -OCH_3_ (*k*_obs_ = 1.78 × 10^−3^ s^−1^). The Hammett treatments of relative reactivities (log *k*_rel_) versus Hammett σ_p_ constants in the hydroxylation reactions gave ρ-values of +0.92 (Figure 10b), which is further evidence of the electrophilic behavior of the (PBI)_2_(4R-Py)Fe^III^OIPh) intermediates. This value is almost identical to the results observed for the pyridine-containing stoichiometric (ρ = +0.87) and catalytic reactions (ρ = +0.97), and is comparable for the 4R-PhI(OAc)_2_-containing catalytic processes (ρ = +0.77).

## 3. Materials and Methods

All materials including 4-substituted pyridines (4-R-Py; R = 4-CH_3_, 4-H, 4-C(O)Ph, 4-C(O)CH_3_, 4-CN), PBI ligand, PhI(OAc)_2_, and substrates (Ph_3_CH) were obtained from Aldrich Chemical Co. and used without further purification unless otherwise noted. Solvents (CH_3_CN, pyridines) were purified according to published procedures, distilled, and stored under argon. [Fe(PBI)_3_](OTf)_2_ was synthesized according to methods in the literature [54]. The UV–Visible spectra were obtained with an Agilent 8453 (Budapest, Hungary) diode-array spectrophotometer using quartz cells. GC and GC-MS analyses were performed on an Agilent 7820A (Budapest, Hungary) equipped with a flame ionization detector and a 30 m Equity-1 column, and a Shimadzu QP2010SE (Budapest, Hungary) equipped with a secondary electron multiplier detector with a conversion dynode and a 30 m HP5MS column, respectively. Cyclic voltammetric experiments were carried out using an SP-150 potentiostat, using EC-Lab V11.41 software. During the measurements we used a three-electrode setup: we used a 3.0 mm diameter glassy-carbon electrode as a working electrode, a Pt wire as counter electrode, and an Ag/AgNO_3_ reference-electrode (https://www.biologic.net/accessory/small-reference-electrodes/; https://www.biologic.net/documents/re-7n-re-7sn-internal-solution-preparation-2/; 12 January 2024) with an acetonitrile solution containing 0.01 mol/dm^3^ silver(I)nitrate (AgNO_3_) and 0.1 mol/dm^3^ tetrabutylammonium perchlorate. The potentials were referred to the Fc/Fc^+^ couple.

*Ligand exchange and stoichiometric oxidation reactions by UV-Vis spectroscopy*: 

All reactions were performed in a 1.0 cm UV cuvette and followed by monitoring UV–Vis spectral changes (increase for the formation of [(PBI)_2_(CH_3_CN)Fe^IlI^OIPh-4R]^3+^ at 760 nm, and decrease in [(PBI)_2_(CH_3_CN)Fe^IlI^OIPh-4R]^3+^ or [(PBI)_2_(4R-Py)Fe^IlI^OIPh]^3+^ in the stoichiometric hydroxylation of triphenylmethane at 700–760 nm) in the reaction solutions. Rate constants (*k*_obs_) were determined under pseudo-first-order conditions (e.g., [Ph_3_CH]/[1] > 100 or [4R-PhI(Oc)_2_]/[1] > 10) by fitting the absorbance changes at 700–760 nm in CH_3_CN at 293 K). Reactions were run at least in triplicate, and the data reported are the average of the reactions. 

*Stoichiometric reaction*: In a typical reaction, [Fe^II^(PBI)_3_](OTf)_2_ (1 ×10^−3^ M) was dissolved in acetonitrile (2 mL), and/or (diacetoxyiodo)benzene (1.2 × 10^−3^ M) derivatives in acetonitrile was added to generate the reactive [(PBI)_2_(CH_3_CN)Fe^IlI^OIPh-4R]^3+^ species. To generate the [(PBI)_2_(4R-Py)Fe^IlI^OIPh]^3+^ species, 10 equivalents of 4R-Py (1 × 10^−2^ M) was added to the [(PBI)_2_(CH_3_CN)Fe^IlI^OIPh]^3+^ intermediate. After the full formation of [(PBI)_2_(CH_3_CN)Fe^IlI^OIPh-4R]^3+^ or [(PBI)_2_(4R-Py)Fe^IlI^OIPh]^3+^, triphenylmethane (1 × 10^−1^ M) was added to the solution. The reactions were monitored with a UV–Vis spectrophotometer at 700–760 nm at 293 K. Reaction rates (*k*_obs_) were calculated using Biochemical Analysis Software (version B.05.01) for Agilent ChemStation.

*Catalytic oxidations:* [Fe^II^(PBI)_3_](OTf)_2_ (1 × 10^−3^ M) was dissolved in acetonitrile (3 mL), and (diacetoxyiodo)benzene derivatives (1 × 10^−1^ M), substrate (3 × 10^−1^ M) (triphenylmethane), and/or different *para*-substituted pyridines (1 × 10^−2^ M) were added to the solution. Bromobenzene (1 × 10^−1^ M) was used as an internal standard. The mixture was stirred for 4 h at 323 K, the solution was freed from the metal catalyst using an Al_2_O_3_-filled column, the products from the diluted solutions were identified by GC-MS, and the yields were determined by GC.

Product of triphenylmethane oxidation: triphenylmethanol’s GC-MS: (*m*/*z*) relative intensities were 260.10 (M^+^, 14.07); 184.05 (9.15); 183.05 (63.15); 182.10 (15.27); 165.10 (10.26); 155.10 (17.23); 154.10 (28.54); 106.10 (10.08); 105.05 (100); 77.05 (69.30); and 51.05 (16.62).

## 4. Conclusions

**Scheme 2:**
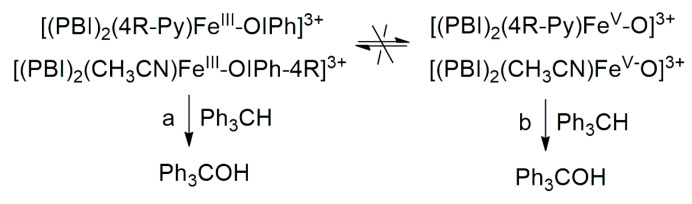
Proposed mechanistic pathways for the iron(III)–iodosylarene ([(PBI)_2_[(4R-Py)Fe^III^(OIPh-4R)]^3+^) mediated oxidation of triphenylmethane.

The aim of this work was to generate an iodosylarene–iron(III) ([(PBI)_2_(CH_3_CN)Fe^IlI^OIPh]^3+^) intermediate with a labile site, thus the effect of nitrogen-containing co-ligands (4R-Py, R = -CH_3_, -H, C(O)CH_3_, -C(O)Ph, -CN) for their spectral properties and reactivity in CH-activation reactions can be tested by ligand exchange reactions. In a similar way, we wanted to investigate the effect of substituted 4R’-PhI(OAc)_2_ (R’ = -OMe, -Me, -H, -Cl, -C(O)Me) derivatives as oxidizing agents in both stoichiometric and catalytic oxidation reactions towards triphenylmethane. It was found that both pyridine and iodosylbenzene derivatives as ligands (oxidants) can induce a hypsochromic shift in the complexes [(PBI)_2_(4R-Py)Fe^IlI^OIPh]^3+^ and [(PBI)_2_(CH_3_CN)Fe^IlI^OIPh-4R’]^3+^, which is consistent and proportional to the electron density of the metal center. The linear correlation between λ_max_^−1^ (at 700–760 nm) and the Hammett constants (σ_p_) of [(PBI)_2_(4R-Py)Fe^IlI^OIPh]^3+^ indicates that the observed hypsochromic shift (blue shift) of the absorption bands can be attributed to the electronic effect of the co-ligands (4R-Py) and the electrophilicity of the active intermediate. Based on Hammett correlations between the log*k*_rel_ and the σ_p_ (4R-Py and 4R’-PhI(OAc)_2_) parameters, strong evidence for the electrophilic nature of the reactive species was observed for both stoichiometric (ρ = +0.87 and +0.92, respectively) and catalytic (ρ = +0.97 and +0.77, respectively) reactions. Furthermore, the redox potential values of [(PBI)_2_(4R-Py)Fe^IlI^OIPh]^3+^ intermediates correlate very well with the hydroxylation rate of the triphenylmethane. The highest reactivity increase was achieved by the intermediates [(PBI)_2_(4R-Py)Fe^IlI^OIPh]^3+^ and [(PBI)_2_(CH_3_CN)Fe^IlI^OIPh-4R’]^3+^ containing an electron-withdrawing group. Based on the competitive experiments, similar results were observed in both the catalytic and stoichiometric reactions, which suggests that the electrophilic [(PBI)_2_(4R-Py)Fe^III^OIPh-4R’]^3+^ intermediate is formed as the reactive species responsible for the oxidation reactions. These results clearly indicate that the reactive form of the hydroxylation reactions is the iron(III)–iodosylarene adduct (Scheme 2, Pathway a), and not the iron(V)oxo complex (Scheme 2 Pathway b).

## Figures and Tables

**Table 1:** [Fe^II^(PBI)_3_](OTf)_2-_catalyzed hydroxylation of triphenylmethane with PhI(OAc)_2_ in the presence of *para*-substituted pyridines in acetonitrile at 323 K ^1^.

Entry	Co-Ligand	Conversion (%) ^2,3^	TOF (1/h) ^4^	*k*_rel_ ^5^
1	-	24.71	6.17	-
2	4-CH_3_-Py	19.98	4.99	0.48
3	Py	37.12	9.28	1.00
4	4-C(O)C_6_H_5_-Py	59.96	14.99	1.97
5	4-C(O)CH_3_-Py	63.55	15.88	2.17
6	4-CN-Py	83.00	20.75	3.82

^1^ Reaction conditions: see experimental section. ^2^ The product was identified by GC-MS and the yields (based on oxidant) were determined by GC using bromobenzene as internal standard. ^3^ Conversion = TON ([Substrate]_0_/[**1**]_0_). ^4^ TON / h. ^5^ k_rel_ = log(X_f_/X_i_)/log(Y_f_/Y_i_), where X_i_ and X_f_ are the initial and final concentrations of Ph_3_CH in the presence of 4R-Py, and Y_i_ and Y_f_ are the initial and final concentrations of Ph_3_CH in the presence of Py. Reaction conditions: [**1**]_0_ = 1 × 10^−3^ M, [PhI(OAc)_2_]_0_ = 1 × 10^−1^ M, [Ph_3_CH]_0_ = 3 × 10^−1^ M, [4R-Py]_0_ = 1 × 10^−2^ M.

**Table 2:** Formation and self-decay of [(PBI)_2_(MeCN)Fe^III^(4R’-PhIO)]^3+^ intermediates in the in situ reaction of **1** with 4R-PhI(OAc)_2_ in acetonitrile at 293 K.

Entry	Oxidant	*k*_obs_ (s^−1^) ^1^	*k*_rel_	*k_decay_* (10^−4^ s^−1^)	*t*_1/2_ (s)
1	4- CH_3_O-PhI(OAc)_2_	0.637 ± 0.03	1.55	1.22	9000
2	4- CH_3_-PhI(OAc)_2_	0.436 ± 0.02	1.06	4.27	8300
3	4-H-PhI(OAc)_2_	0.410 ± 0.02	1.00	6.41	6850
4	4-Cl-PhI(OAc)_2_	0.297 ± 0.01	0.72	- ^2^	-
5	4-C(O)CH_3_-PhI(OAc)_2_	0.187 ± 0.01	0.46	- ^2^	-

^1^ Reactions were followed by UV–Vis spectroscopy at 760 nm. Reaction conditions: [[Fe^II^(PBI)_3_](OTf)_2_]_0_ = 1 × 10^−3^ M, [4R-PhI(OAc)_2_]_0_ = 1 × 10^−1^ M in acetonitrile. ^2^ Precipitation was observed.

**Table 3:** [Fe^II^(PBI)_3_](OTf)_2_-catalyzed hydroxylation of triphenylmethane with 4R’-PhI(OAc)_2_ in acetonitrile at 323 K ^1^.

Entry	Oxidant	Conversion (%) ^2,3^	TOF (1/h) ^4^	*k*_rel_ ^5^
1	4-MeO-PhI(OAc) _2_	13.87	3.47	0.53
2	4-Me-PhI(OAc) _2_	17.26	4.32	0.67
3	4-H-PhI(OAc) _2_	24.71	6.17	1.00
4	4-Cl-PhI(OAc) _2_	30.33	7.58	1.27
5	4-C(O)Me-PhI(OAc) _2_	45.75	11.44	2.15

^1^ Reaction conditions: see experimental section. ^2^ The product was identified by GC-MS and the yields (based on oxidant) were determined by GC using bromobenzene as internal standard. ^3^ Conversion = TON ([Substrate]_0_/[**1**]_0_). ^4^ TON/h. ^5^ k_rel_ = log(X_f_/X_i_)/log(Y_f_/Y_i_), where X_i_ and X_f_ are the initial and final concentrations of Ph_3_CH in the presence of 4R’-Ph(IOAc)_2_, and Y_i_ and Y_f_ are the initial and final concentrations of Ph_3_CH in the presence of Ph(IOAc)_2_. Reaction conditions: [**1**]_0_ = 1 × 10^−3^ M, [4R’-PhI(OAc)_2_]_0_ = 1 × 10^−1^ M, [Ph_3_CH]_0_ = 3 × 10^−1^ M.

**Table 4:** Summary of kinetic data for the stoichiometric oxidation of triphenylmethane with [(PBI)_2_Fe^III^(OIPh)(4R-Py)] intermediates generated in situ by the reaction of **1** with Ph(IOAc)_2_ and 4R-Py derivatives in acetonitrile at 293 K ^1^.

Entry	[(PBI)_2_(4R-Py)Fe^IlI^OIPh]^3+^ R=	λ (nm)	ν (cm^−1^)	σ	*k*_obs_ (10^−4^ s^−1^) ^2^	*k*_rel_
1	4-Me-Py	700	14,286	−0.17	8.95 ± 0.41	0.67
2	Py	723	13,831	0	13.31 ± 0.46	1.00
3	4-C(O)C_6_H_5_-Py	753	13,280	0.42	33.77 ± 1.67	2.54
4	4-C(O)CH_3_-Py	748	13,369	0.5	36.96 ± 1.48	2.78
5	4-CN-Py	754	13,263	0.66	41.60 ± 1.25	3.13
6	PyO	750	13,333	-	31.40 ± 0.97	-

^1^ Reaction conditions: [**1**]_0_ = 1 × 10^−3^ M, [PhI(OAc)_2_]_0_ = 1.2 × 10^−3^ M, [triphenylmethane]_0_ = 1 × 10^−1^ M, [4R-Py]_0_ = 1 × 10^−2^ M. ^2^ Reactions were followed by UV–Vis spectroscopy at 700–760 nm.

**Table 5:** Redox properties (CV data) of the [(PBI)_2_Fe^III^(OIPh)(4R-Py)] intermediates generated in situ by the reaction of **1** with Ph(IOAc)_2_ in acetonitrile at 293 K ^1^.

Entry	[(PBI)_2_(4R-Py)Fe^IlI^OIPh]^3+^ R=	*E*_pc_ vs. Fc/Fc^+^ (mV)	*E*_pa_ vs. Fc/Fc^+^ (mV)	*E*_1/2_ vs. Fc/Fc^+^ (mV)
1	4-Me-Py	−396.4	−303.8	−350.0
2	Py	−482.8	−398.0	−440.4
3	4-C(O)C_6_H_5_-Py	−531.2	−470.1	−500.6
4	4-C(O)CH_3_-Py	−549.4	−497.1	−523.2
5	4-CN-Py	−550.1	−497.8	−524.0
6	^-^^2^	−560.0	−495.0	−527.5

^1^ Conditions: [**1**]_0_ = 1 × 10^−3^ M, [PhI(OAc)_2_]_0_= 2 × 10^−3^ M, [4R-Py]_0_ = 1 × 10^−2^ M, in (0.1 M TBAClO_4_) MeCN (10 cm^3^), scan rate: 1500 mV/s. ^2^ Without pyridines with CH_3_CN as co-ligand.

**Table 6:** Stoichiometric hydroxylation of triphenylmethane with [(PBI)_2_(MeCN)Fe^III^(4R-PhIO)]^3+^ intermediates generated in situ by the reaction of **1** with 4R-Ph(IOAc)_2_ oxidants in acetonitrile at 293 K ^1^.

Entry	[(PBI)_2_(CH_3_CN)Fe^IlI^OIPh-R]^3+^ R=	*k*_obs_ (10^−3^ s^−1^)	*k*_rel_
1	4-MeO-PhI(OAc)_2_	1.78 ± 0.05	0.38
2	4-Me-PhI(OAc)_2_	2.87 ± 0.13	0.61
3	4-H-PhI(OAc)_2_	4.70 ± 0.19	1.00
4	4-Cl-PhI(OAc)_2_	7.99 ± 0.33	1.70
5	4-C(O) Me-PhI(OAc)_2_	9.22 ± 0.52	1.96

^1^ Reaction conditions: [**1**]_0_= 1 × 10^−3^ M, [4R-PhI(OAc)_2_]_0_ = 1.2 × 10^−3^ M, [Ph_3_CH]_0_ = 1 × 10^−1^ M in acetonitrile.

## Data Availability

Data is contained within the article or Supplementary Materials.

## Supplementary Materials

The following supporting information can be downloaded at: https://www.mdpi.com/article/10.3390/molecules29163842/s1.
