# Supplementary material for: Effect of Substituted Pyridine Co-Ligands and (Diacetoxyiodo)benzene Oxidants on the Fe(III)-OIPh-Mediated Triphenylmethane Hydroxylation Reaction
Source: Molecules. 2024 Aug 13;29(16):3842. doi: 10.3390/molecules29163842 (PMC11357111; doi:10.3390/molecules29163842)
Supplement: Supplementary file 1 [file molecules-29-03842-s001.zip › molecules-3133653-supplementary.pdf]

Supporting Information

Effect of substituted pyridine co-ligands and (diacetoxy-iodo)benzene oxidants on the Fe(III)-OIPh mediated triphenylmethane hydroxylation reaction

Patrik Török and József Kaizer *

Research Group of Bioorganic and Biocoordination Chemistry, University of Pannonia, H-8201 Veszprém, Hungary; patriktrk6@gmail.com (P.T.)

* Correspondence: kaizer@almos.uni-pannon.hu

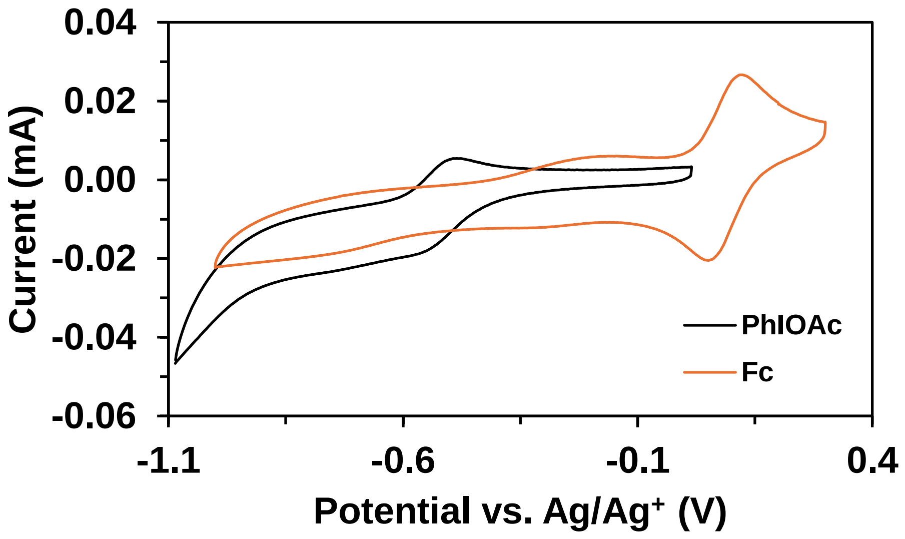

Figure S1. $[1]_0 = 1.0\times10^{-3}$ M, $[4H\text{-}PhI(OAc)_2]_0 = 2.0\times10^{-3}$ M, $[Ferrocene]_0 = 2.0\times10^{-3}$ M, in (0.1 M $TBAClO_4$) MeCN (10 cm^3), scan rate: 1500 mV/s

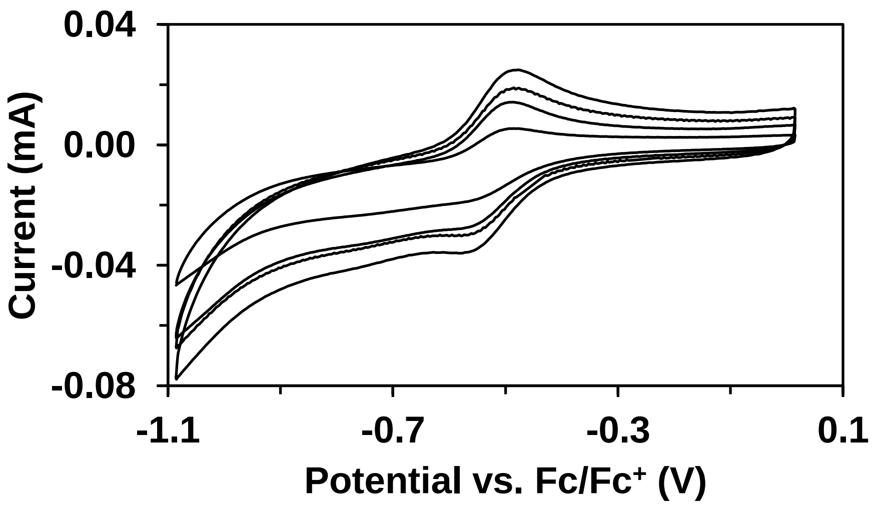

Figure S2. $[1]_0 = 1.0\times10^{-3}$ M, $[4\text{-}H\text{-}PhI(OAc)_2]_0 = 2.0\times10^{-3}$ M, in (0.1 M $TBAClO_4$) MeCN (10 cm^3), scan rate: 500, 1000, 1500, 2000 mV/s

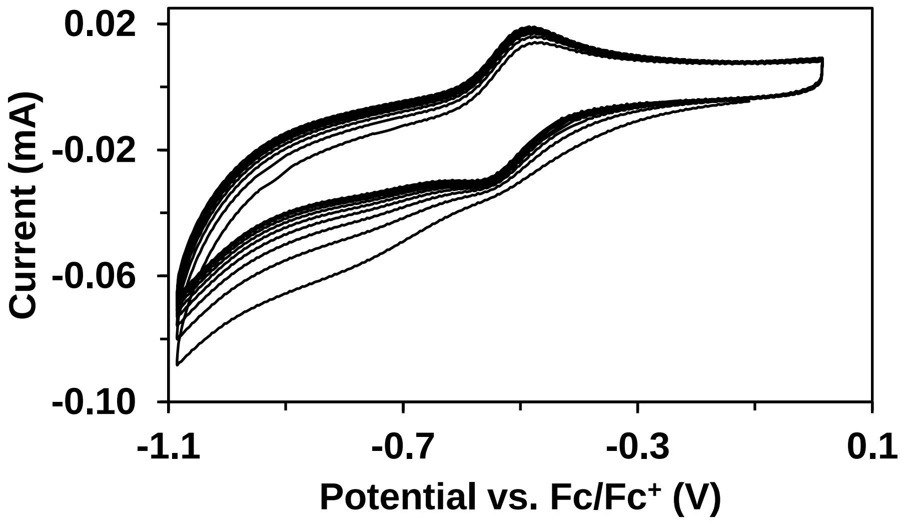

Figure S3. $[1]_0 = 1.0\times10^{-3}$ M, $[4\text{-H-PhI(OAc)}_2]_0 = 2.0\times10^{-3}$ M, in (0.1 M $TBAClO_4$) MeCN (10 cm^3), scan rate: 1500 mV/s, cycles: 10

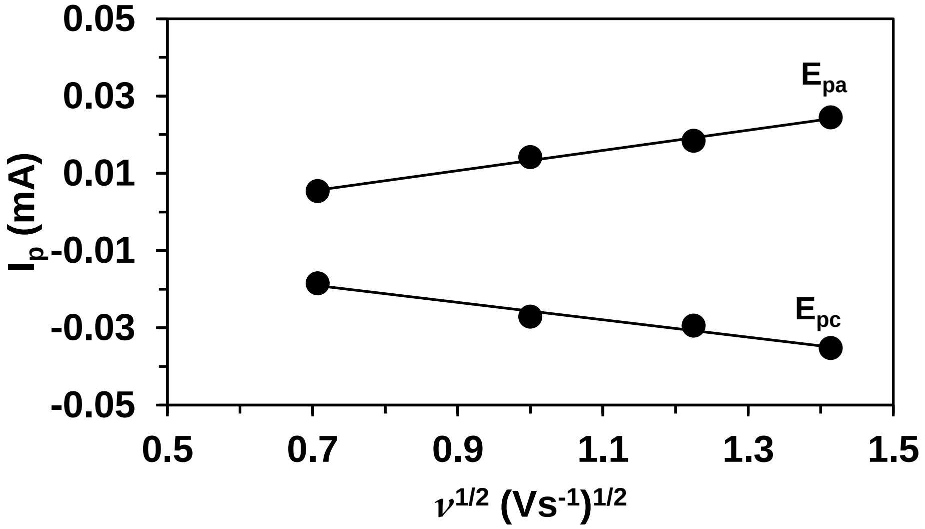

Figure S4. Randles-Sevcik plot of $[1]_0 = 1.0\times10^{-3}$ M, $[4\text{-H-PhI(OAc)}_2]_0 = 2.0\times10^{-3}$ M, in (0.1 M $TBAClO_4$) MeCN (10 cm^3), scan rate: 500-2000 mV/s

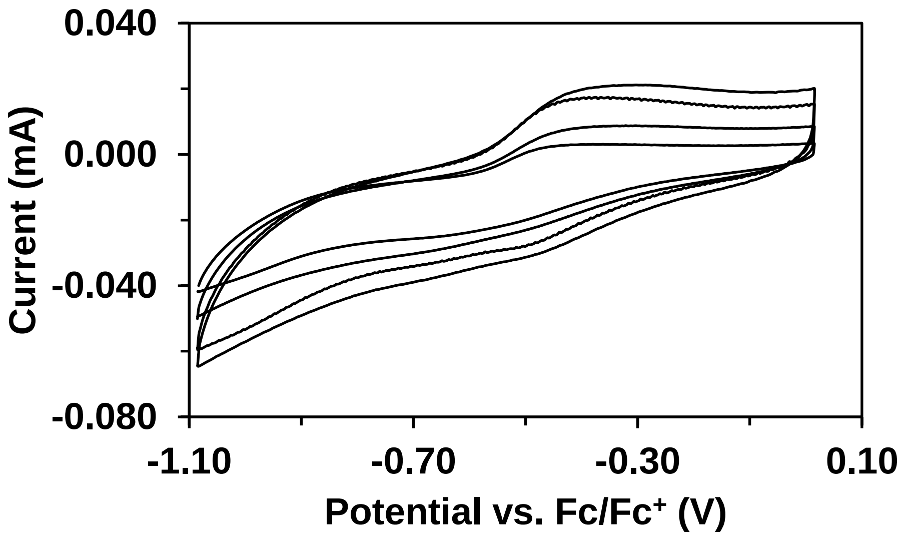

Figure S5. $[1]_0 = 1.0\times10^{-3}$ M, $[\text{4-H-PhI(OAc)}_2]_0 = 2.0\times10^{-3}$ M, $[\text{Py}]_0 = 10.0\times10^{-3}$ M, in (0.1 M $TBAClO_4$) MeCN (10 cm^3), scan rate: 500, 1000, 1500, 2000 mV/s

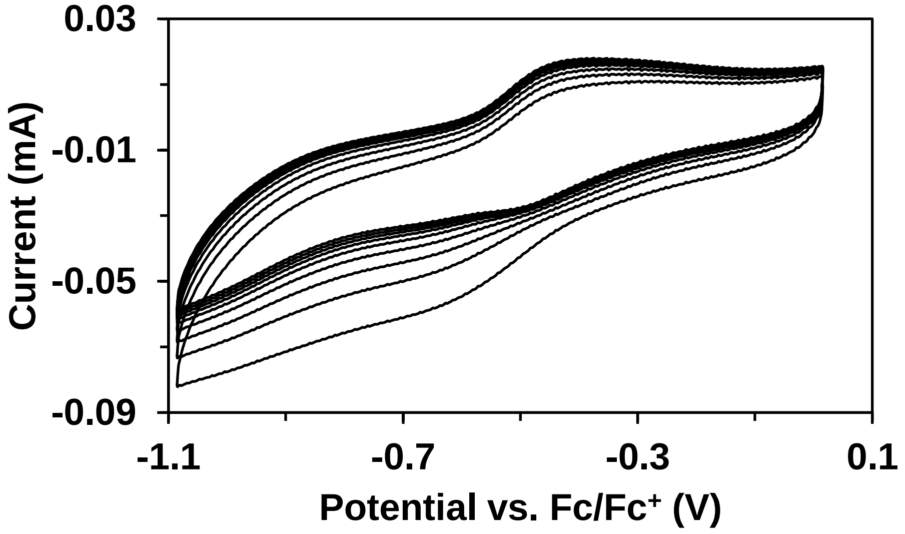

Figure S6. $[1]_0 = 1.0\times10^{-3}$ M, $[\text{4-H-PhI(OAc)}_2]_0 = 2.0\times10^{-3}$ M, $[\text{Py}]_0 = 10.0\times10^{-3}$ M, in (0.1 M $TBAClO_4$) MeCN (10 cm^3), scan rate: 1500 mV/s, cycles: 10

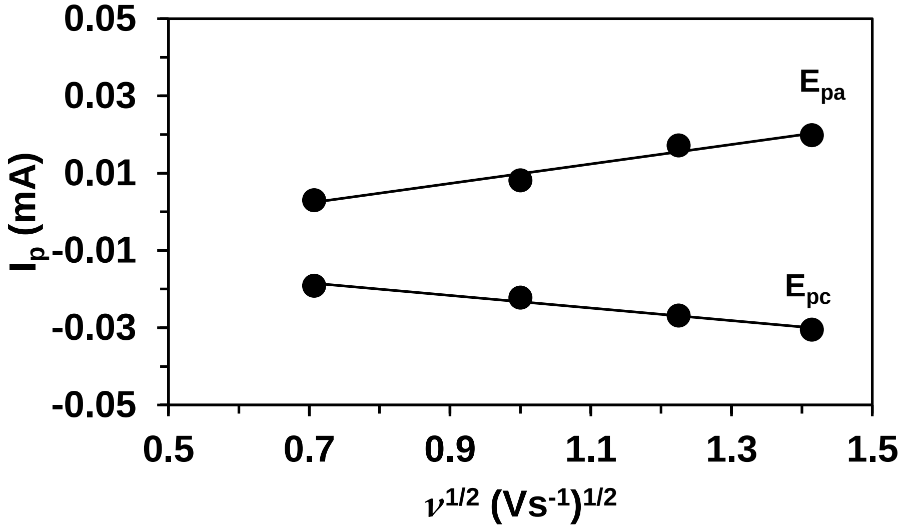

Figure S7. Randles-Sevcik plot of $[1]_0 = 1.0\times10^{-3}$ M, $[4\text{-H-PhI(OAc)}_2]_0 = 2.0\times10^{-3}$ M, $[\text{Py}]_0 = 10.0\times10^{-3}$ M, in (0.1 M $TBAClO_4$) MeCN (10 cm^3), scan rate: 500-2000 mV/s

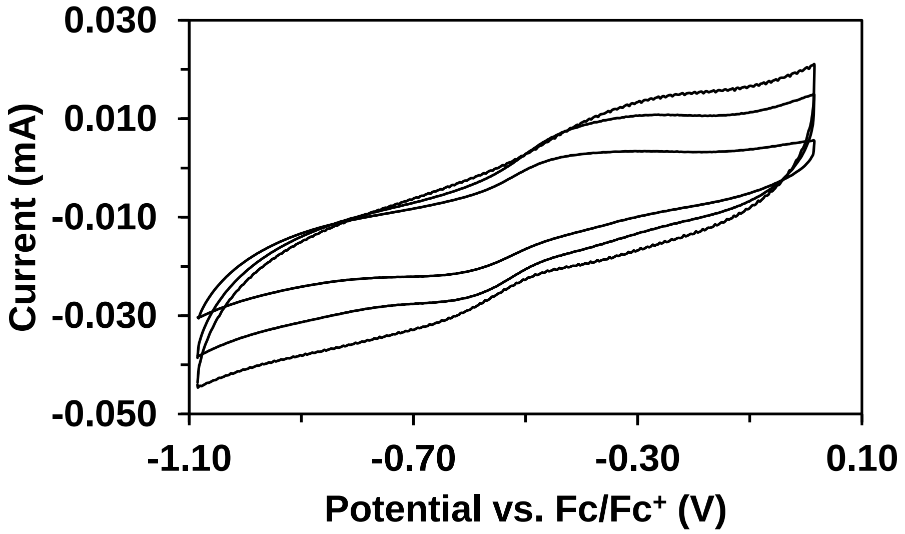

Figure S8. $[1]_0 = 1.0\times10^{-3}$ M, $[4\text{-H-PhI(OAc)}_2]_0 = 2.0\times10^{-3}$ M, $[4\text{-Me-Py}]_0 = 10.0\times10^{-3}$ M, in (0.1 M $TBAClO_4$) MeCN (10 cm^3), scan rate: 500, 1000, 1500 mV/s

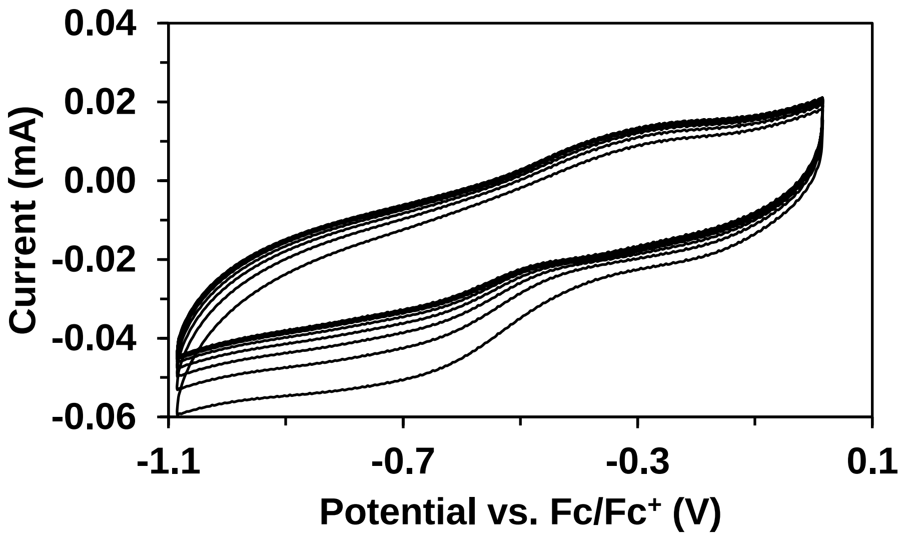

Figure S9. $[1]_0 = 1.0\times10^{-3}$ M, $[4\text{-H-PhI(OAc)}_2]_0 = 2.0\times10^{-3}$ M, $[4\text{-Me-Py}]_0 = 10.0\times10^{-3}$ M, in (0.1 M $TBAClO_4$) MeCN (10 cm^3), scan rate: 1500 mV/s, cycles: 10

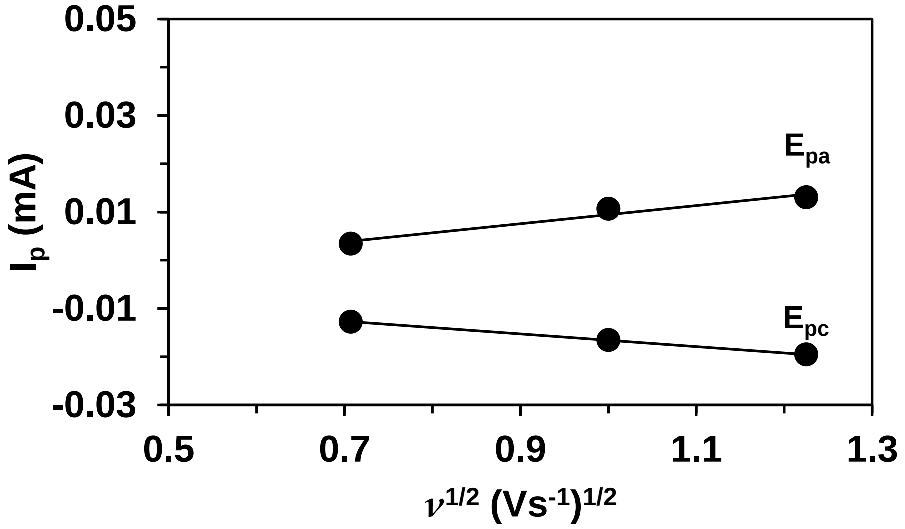

Figure S10. Randles-Sevcik plot of $[1]_0 = 1.0\times10^{-3}$ M, $[4\text{-H-PhI(OAc)}_2]_0 = 2.0\times10^{-3}$ M, $[4\text{-Me-Py}]_0 = 10.0\times10^{-3}$ M, in (0.1 M $TBAClO_4$) MeCN (10 cm^3), scan rate: 500-1500 mV/s

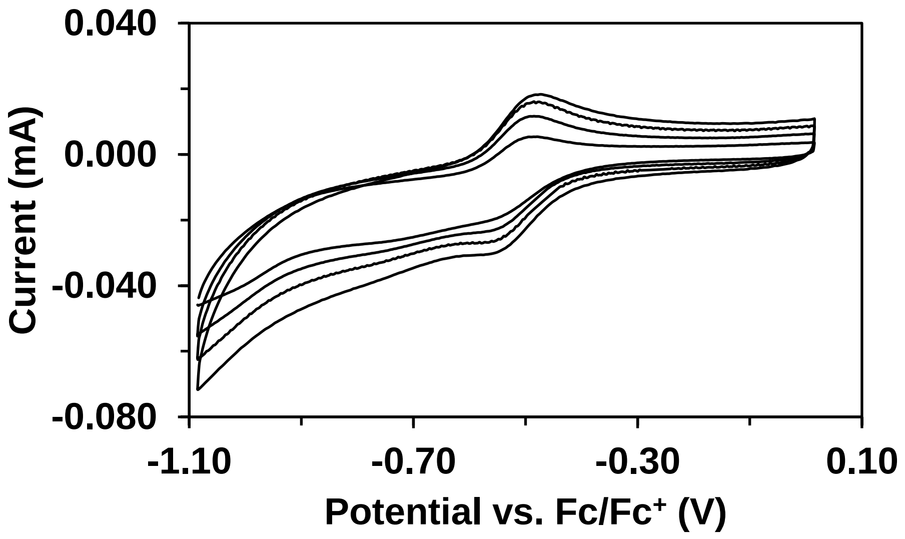

Figure S11. $[1]_0 = 1.0\times10^{-3}$ M, $[\text{4-H-PhI(OAc)}_2]_0 = 2.0\times10^{-3}$ M, $[\text{4-C(O)CH}_3\text{-Py}]_0 = 10.0\times10^{-3}$ M, in (0.1 M $TBAClO_4$) MeCN (10 cm^3), scan rate: 500, 1000, 1500, 2000 mV/s

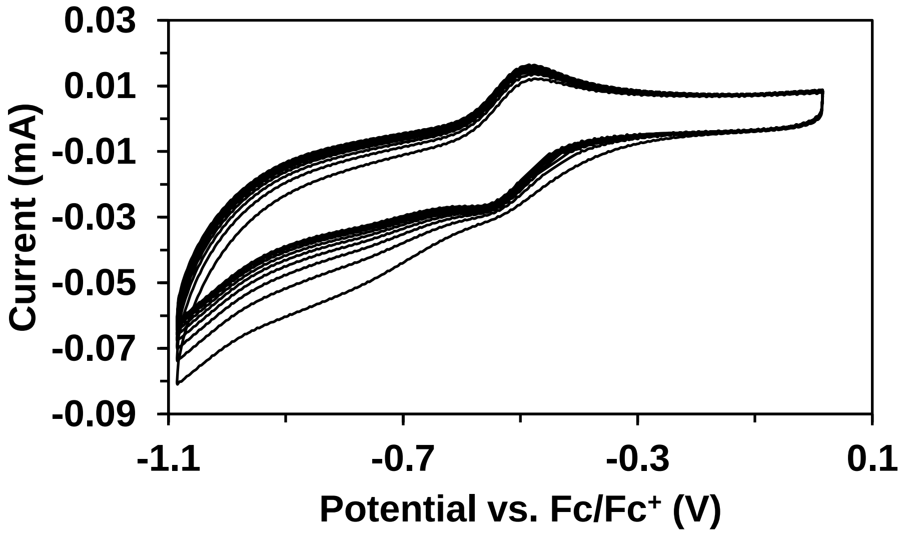

Figure S12. $[1]_0 = 1.0\times10^{-3}$ M, $[\text{4-H-PhI(OAc)}_2]_0 = 2.0\times10^{-3}$ M, $[\text{4-C(O)CH}_3\text{-Py}]_0 = 10.0\times10^{-3}$ M, in (0.1 M $TBAClO_4$) MeCN (10 cm^3), scan rate: 1500 mV/s, cycles: 10

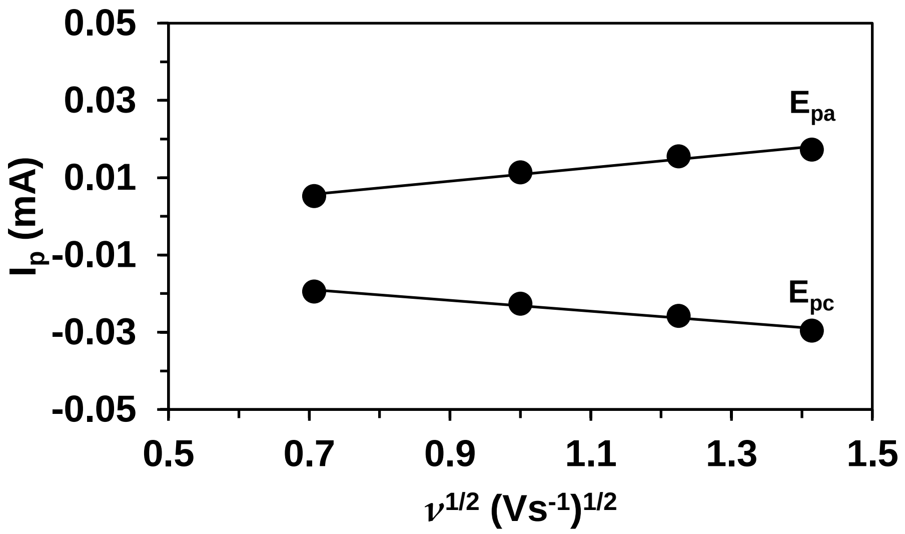

Figure S13. Randles-Sevcik plot of $[1]_0 = 1.0\times10^{-3}$ M, $[4\text{-H-PhI(OAc)}_2]_0 = 2.0\times10^{-3}$ M, $[4\text{-C(O)CH}_3\text{-Py}]_0 = 10.0\times10^{-3}$ M, in (0.1 M $TBAClO_4$) MeCN (10 cm^3), scan rate: 500-1500 mV/s

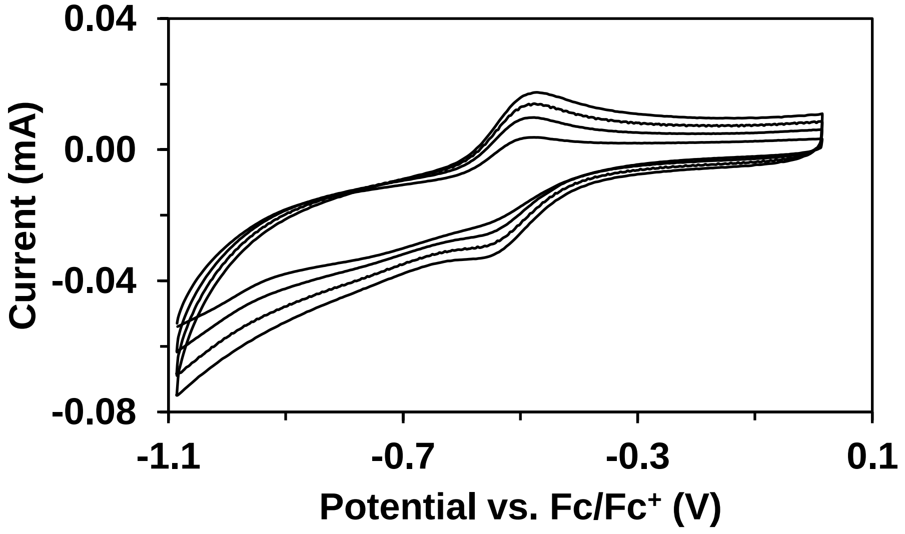

Figure S14. $[1]_0 = 1.0\times10^{-3}$ M, $[4\text{-H-PhI(OAc)}_2]_0 = 2.0\times10^{-3}$ M, $[4\text{-CN-Py}]_0 = 10.0\times10^{-3}$ M, in (0.1 M $TBAClO_4$) MeCN (10 cm^3), scan rate: 500, 1000, 1500, 2000 mV/s

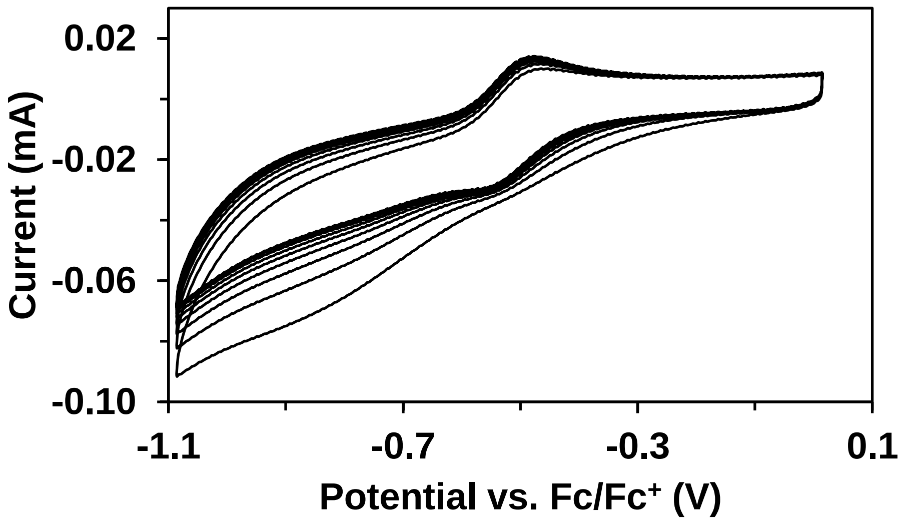

Figure S15. $[1]_0 = 1.0\times10^{-3}$ M, $[\text{4-H-PhI(OAc)}_2]_0 = 2.0\times10^{-3}$ M, $[\text{4-CN-Py}]_0 = 10.0\times10^{-3}$ M, in (0.1 M $TBAClO_4$) MeCN (10 cm^3), scan rate: 1500 mV/s, cycles: 10

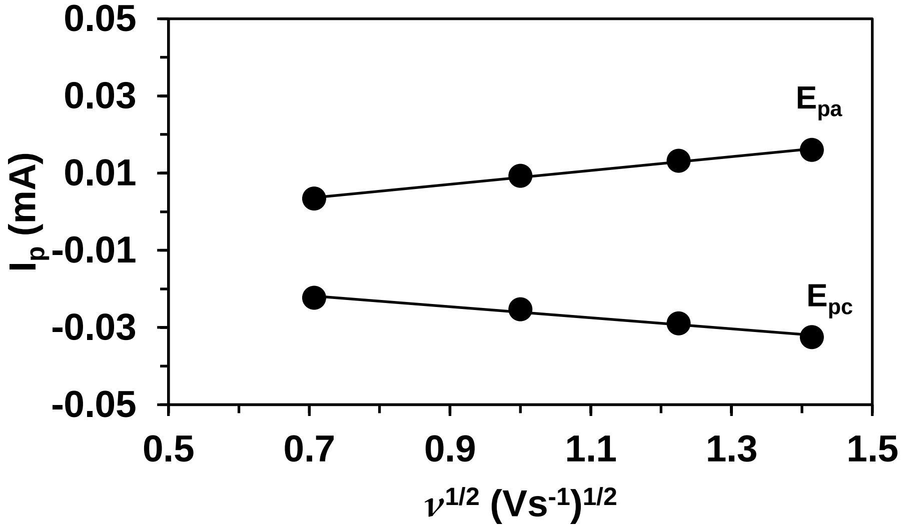

Figure S16. Randles-Sevcik plot of $[1]_0 = 1.0\times10^{-3}$ M, $[\text{4-H-PhI(OAc)}_2]_0 = 2.0\times10^{-3}$ M, $[\text{4-CN-Py}]_0 = 10.0\times10^{-3}$ M, in (0.1 M $TBAClO_4$) MeCN (10 cm^3), scan rate: 500-2000 mV/s

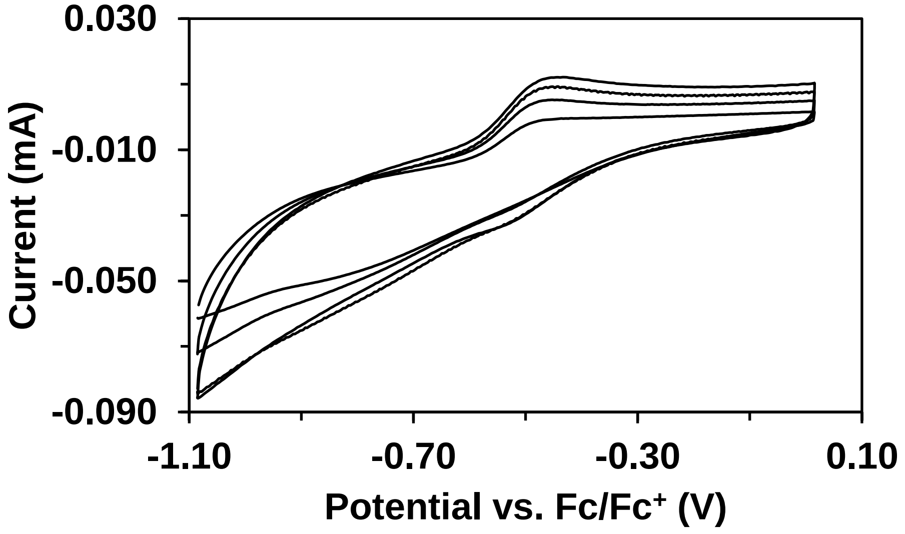

Figure S17. $[1]_0 = 1.0\times10^{-3}$ M, $[4\text{-H-PhI(OAc)}_2]_0 = 2.0\times10^{-3}$ M, $[4\text{-C(O)C}_6\text{H}_5\text{-Py}]_0 = 10.0\times10^{-3}$ M, in (0.1 M $TBAClO_4$) MeCN (10 cm^3), scan rate: 500, 1000, 1500, 2000 mV/s

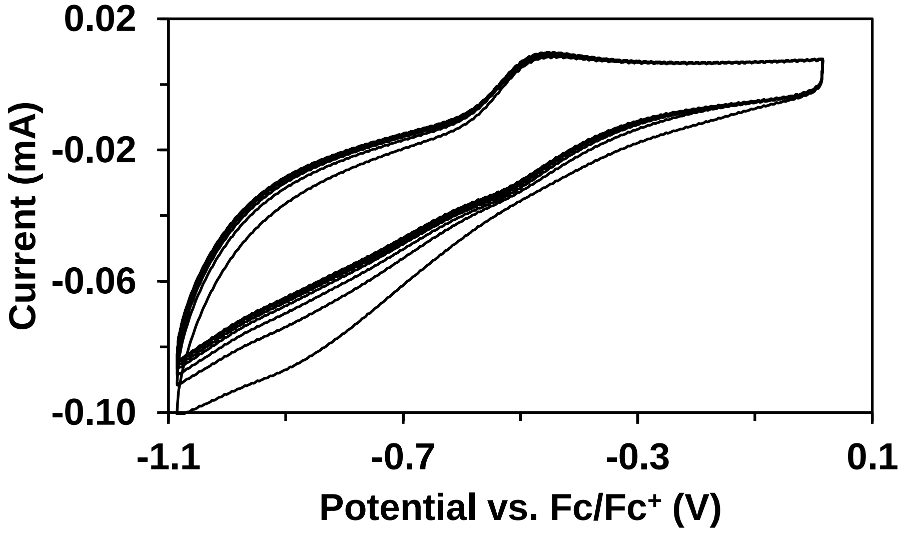

Figure S18. $[1]_0 = 1.0\times10^{-3}$ M, $[4\text{-H-PhI(OAc)}_2]_0 = 2.0\times10^{-3}$ M, $[4\text{-C(O)C}_6\text{H}_5\text{-Py}]_0 = 10.0\times10^{-3}$ M, in (0.1 M $TBAClO_4$) MeCN (10 cm^3), scan rate: 1500 mV/s, cycles: 10

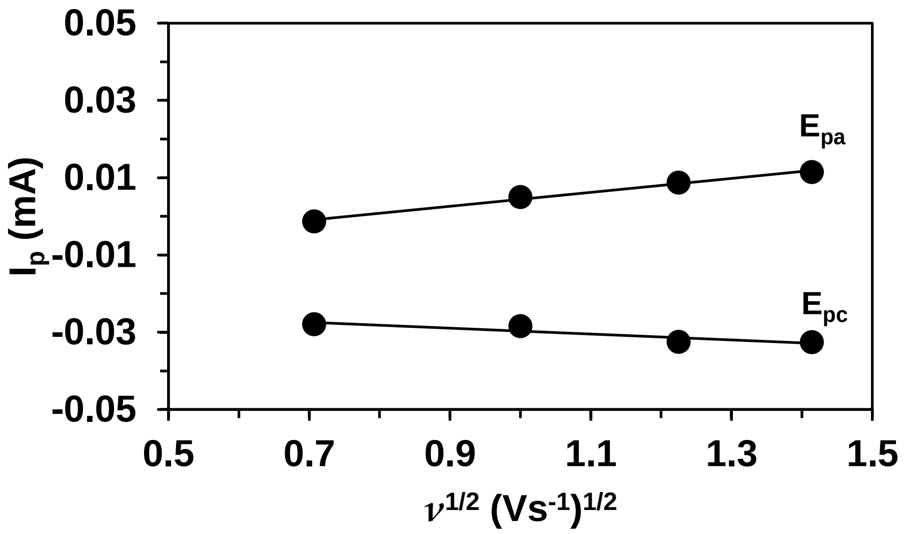

Figure S19. Randles-Sevcik plot of $[1]_0 = 1.0\times10^{-3}$ M, $[4\text{-H-PhI(OAc)}_2]_0 = 2.0\times10^{-3}$ M, $[4\text{-C(O)C}_6\text{H}_5\text{-Py}]_0 = 10.0\times10^{-3}$ M, in (0.1 M $TBAClO_4$) MeCN (10 cm^3), scan rate: 500-1500 mV/s

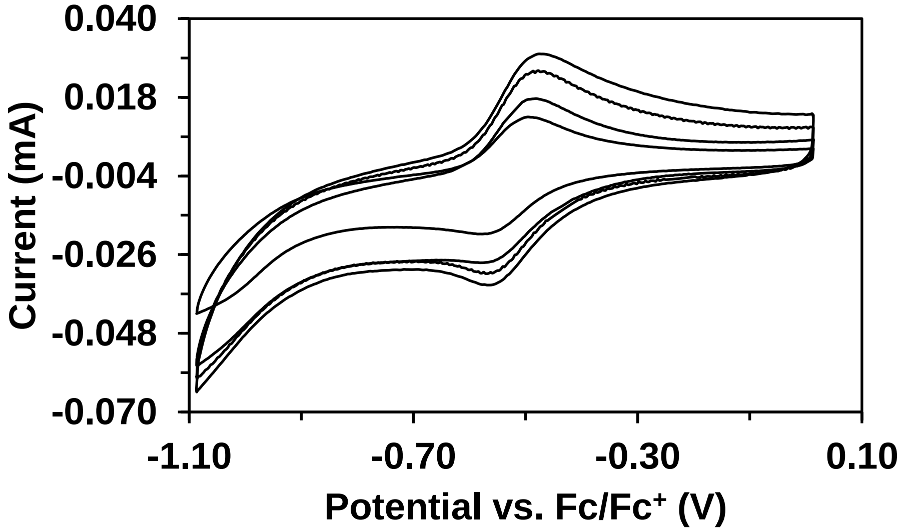

Figure S20. $[1]_0 = 1.0\times10^{-3}$ M, $[4\text{-Cl-PhI(OAc)}_2]_0 = 2.0\times10^{-3}$ M, in (0.1 M $TBAClO_4$) MeCN (10 cm^3), scan rate: 500, 1000, 1500, 2000 mV/s

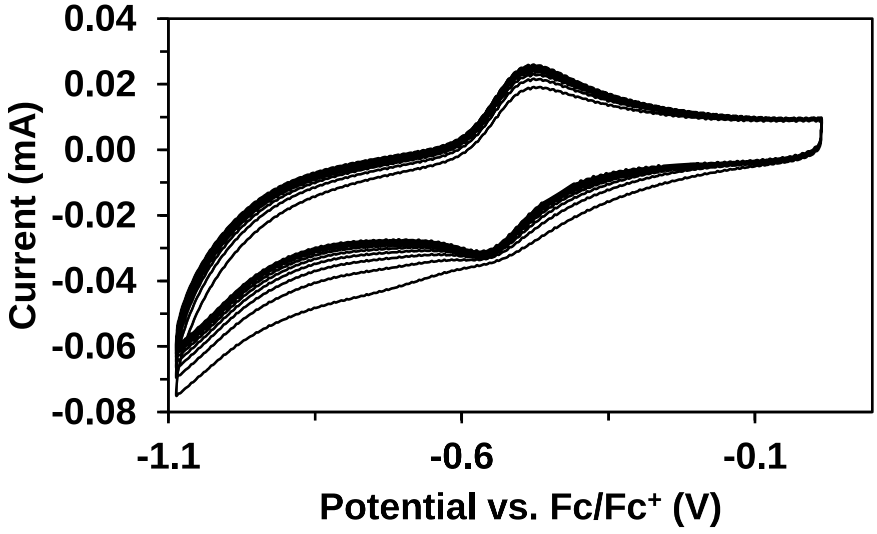

Figure S21. $[1]_0 = 1.0\times10^{-3}$ M, $[4\text{-Cl-PhI(OAc)}_2]_0 = 2.0\times10^{-3}$ M, in (0.1 M $TBAClO_4$) MeCN (10 cm^3), scan rate: 1500 mV/s, cycles: 10

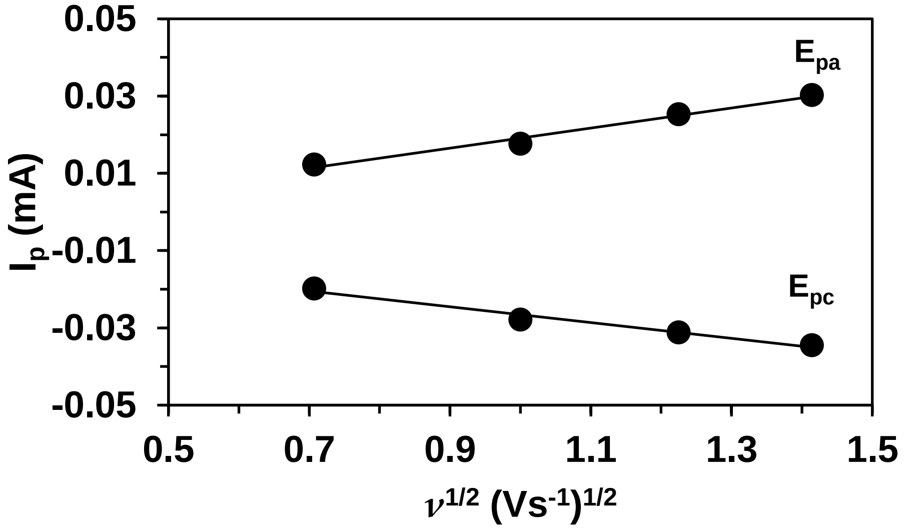

Figure S22. Randles-Sevcik plot of $[1]_0 = 1.0\times10^{-3}$ M, $[4\text{-Cl-PhI(OAc)}_2]_0 = 2.0\times10^{-3}$ M, in (0.1 M $TBAClO_4$) MeCN (10 cm^3), scan rate: 500-2000 mV/s

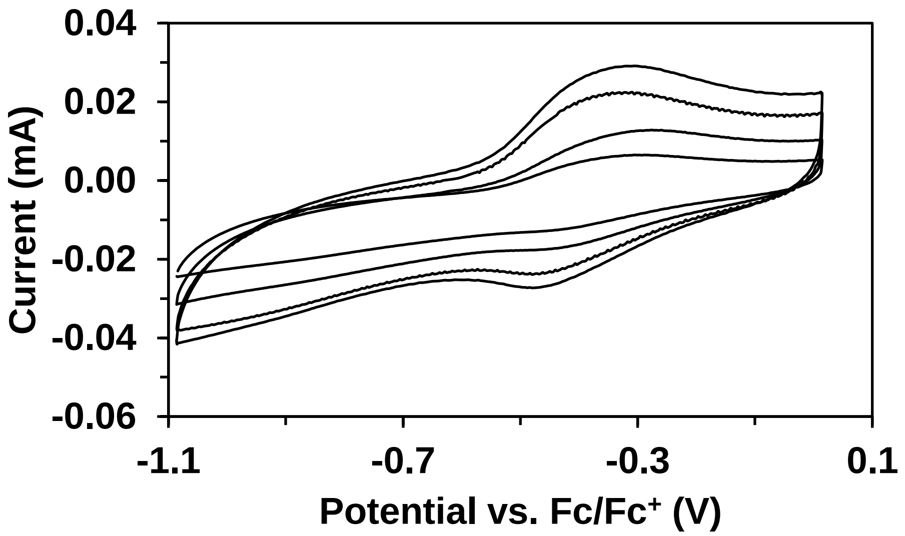

Figure S23. $[1]_0 = 1.0\times10^{-3}$ M, $[4\text{-MeO-PhI(OAc)}_2]_0 = 2.0\times10^{-3}$ M, in (0.1 M $TBAClO_4$) MeCN (10 cm^3), scan rate: 500, 1000, 1500, 2000 mV/s

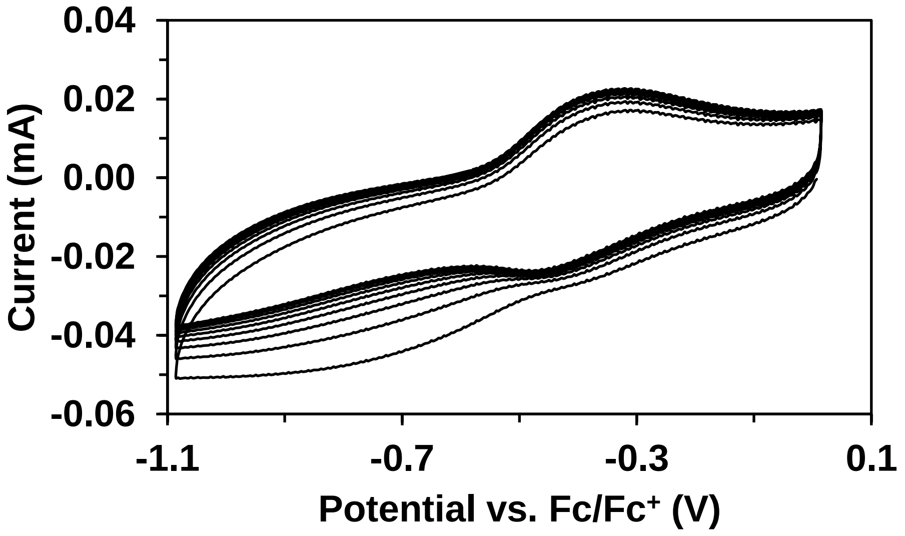

Figure S24. $[1]_0 = 1.0\times10^{-3}$ M, $[4\text{-MeO-PhI(OAc)}_2]_0 = 2.0\times10^{-3}$ M, in (0.1 M $TBAClO_4$) MeCN (10 cm^3), scan rate: 1500 mV/s, cycles: 10

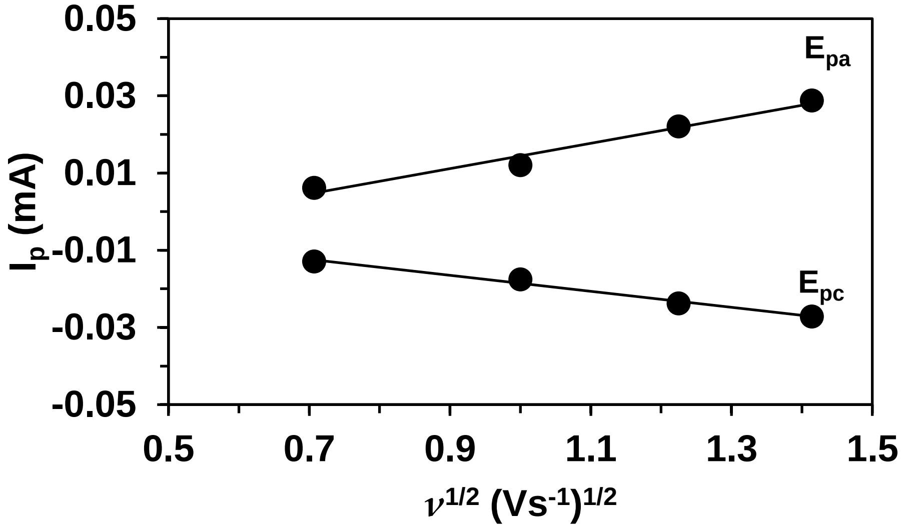

Figure S25. Randles-Sevcik plot of $[1]_0 = 1.0\times10^{-3}$ M, $[4\text{-MeO-PhI(OAc)}_2]_0 = 2.0\times10^{-3}$ M, in (0.1 M $TBAClO_4$) MeCN (10 cm^3), scan rate: 500-2000 mV/s

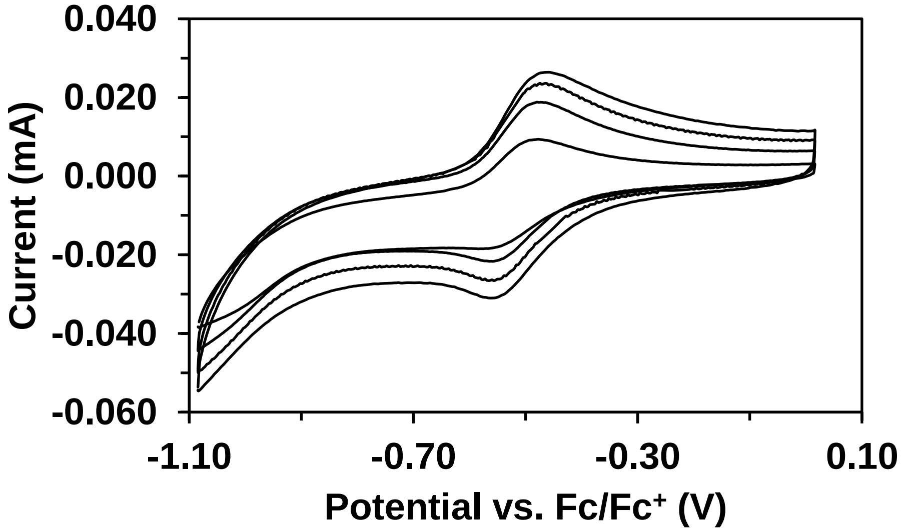

Figure S26. $[1]_0 = 1.0\times10^{-3}$ M, $[4\text{-Me-PhI(OAc)}_2]_0 = 2.0\times10^{-3}$ M, in (0.1 M $TBAClO_4$) MeCN (10 cm^3), scan rate: 500, 1000, 1500, 2000 mV/s

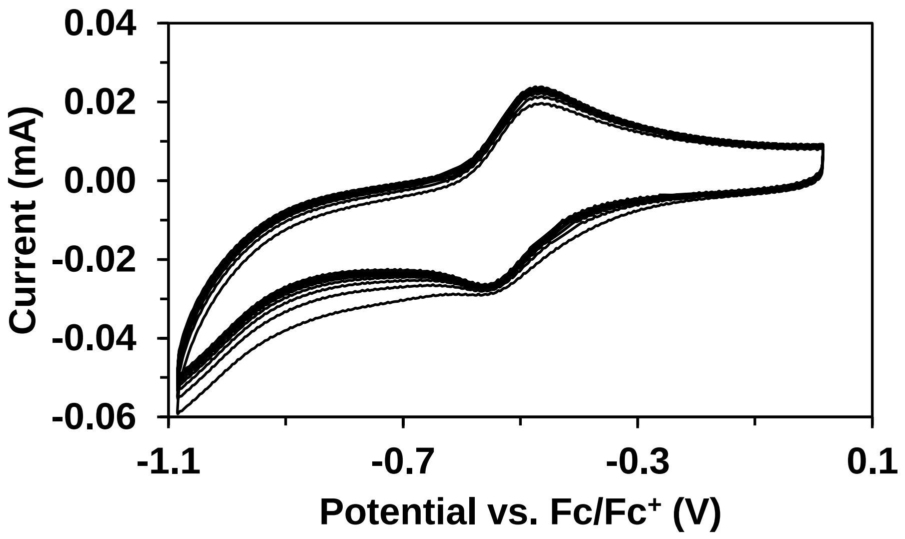

Figure S27. $[1]_0 = 1.0\times10^{-3}$ M, $[4\text{-Me-PhI(OAc)}_2]_0 = 2.0\times10^{-3}$ M, in (0.1 M $TBAClO_4$) MeCN (10 cm^3), scan rate: 1500 mV/s, cycles: 10

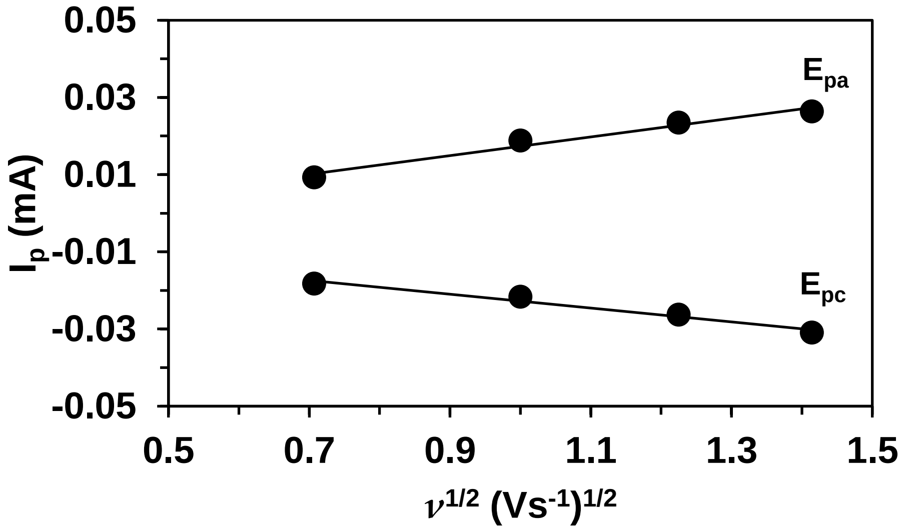

Figure S28. Randles-Sevcik plot of $[1]_0 = 1.0\times10^{-3}$ M, $[4\text{-Me-PhI(OAc)}_2]_0 = 2.0\times10^{-3}$ M, in (0.1 M $TBAClO_4$) MeCN (10 cm^3), scan rate: 500-2000 mV/s

Reference electrode: Ag vire with an acetonitrile solution containing 0.01 mol/dm^3 silver(I)nitrate ($AgNO_3$) and 0.1 mol/dm^3 tetrabutylammonium perchlorate.

https://www.biologic.net/accessory/small-reference-electrodes/

https://www.biologic.net/documents/re-7n-re-7sn-internal-solution-preparation-2/
